# “Multilocus sequence analysis for population diversity of indigenous entomopathogenic fungus *Beauveria bassiana* and its bio-efficacy against the cassava mite, *Tetranychus truncatus* Ehara (Acari: Tetranychidae)”

**DOI:** 10.3389/fmicb.2022.1007017

**Published:** 2022-10-11

**Authors:** M. Chaithra, T. Prameeladevi, S. N. Bhagyasree, L. Prasad, S. Subramanian, Deeba Kamil

**Affiliations:** ^1^Division of Plant Pathology, ICAR-Indian Agricultural Research Institute, New Delhi, India; ^2^Division of Entomology, ICAR-Indian Agricultural Research Institute, New Delhi, India

**Keywords:** *Beauveria bassiana*, morphological characteristics, molecular identification, multigene phylogenetics diversity, *Tetranychus truncatus*

## Abstract

*Beauveria bassiana* is an entomopathogenic fungus that causes the white muscadine disease in insects. The majority of entomopathogenic fungi are soil and insect borne, 15 soil samples were collected from seven different locations during 2021, from January to December. Similarly, during 2022, March to December, 15 fungus-infected insect specimens were collected from five different locations hence soil and insect samples from various ecosystems were collected. As a result, 30 *B. bassiana* isolates from 11 different geographical areas were identified using morphological characteristics and multilocus sequence data in this investigation. The taxonomical positions of the isolates were determined using morphological characteristics and phylogenetic inferences based on three loci (Internal Transcribed Sequence, Elongation Factor-1α, and *B. bassiana* chitinase 1). In phylogenetic analysis of *B. bassiana*, the Maximum Likelihood analytical method produced distinct tree topology when compared to Neighbor-joining and minimum evolution. Three isolates *viz.*, Bb3, Bb7 and Bb20 were found closely linked with reference isolate (KTU-24) and other showed the higher population diversity among them. The genetic distances of 30 *B. bassiana* isolates revealed that 15 were not closely related (D varied from 0.003 to 0.036). The pathogenicity of *B. bassiana* isolates from various hosts along with one commercial formulation (Beveroz) was assessed against *Tetranychus truncatus* under *in vitro* conditions by a completely randomized design (CRD) experiment. The same experiment was repeated thrice to confirm the pathogenicity of *B. bassiana* against *T. truncatus*. Later, the collected *T. truncatus* mortality data was converted into corrected mortality by using the Abbott formula and the values were examined using analysis of variance (ANOVA) in SPSS 23.0 software. Duncan’s Multiple Comparison Test was also done to compare the percentage mortality rates among the 30 *B. bassiana* isolates. The recorded results showed that the Bb6, Bb15 and Bb12 isolates caused significantly higher mortality of *T. truncatus*, i.e., 97.73, 96.73 and 94.50% respectively, than the other isolates. This study showed the relativeness among the *B. bassiana* isolates and establishes their bio-efficacy against *T. truncatus*, which further can be used for commercialization as bio-pesticide.

## Introduction

*Beauveria* is the most prevalent entomopathogenic fungal genus worldwide (Robene-Soustrade et al., 2015; Chen et al., 2018). It had broad host range (Inglis et al., 2001) and can be isolated from insects, mites, and soil in all parts of the world (Boucias and Pendland, 1998). *Beauveria bassiana* populations are genetically and phenotypically diverse, range from sympodial to whorled clusters of short-globose to flask-shaped conidiogenous cells, which give rise to a succession of one-celled, hyaline, holoblastic conidia carried on a progressively elongated sympodial rachis, morphological differentiation. In *Beauveria*, conidia are the most important morphological trait for species identification. Conidia are globose, ellipsoidal, reniform to cylindrical or comma shaped and 1.7 to 5.5 μm in diameter in size. However, phenotypic traits are insufficient to identify *Beauveria* isolates or to monitor biocontrol agent releases in the field (Castrillo et al., 2003). As a result, a number of genetic approaches have been developed to address taxonomy and genetic variation issues in *B. bassiana.*

*B. bassiana* genetic diversity, characterization, and relatedness were successfully studied using molecular markers. Grasp population structure, gene flow, isolate type, ecology, and possible impact when utilized as insect biological control agents requires a thorough understanding of genetic diversity and intraspecies relationships. For molecular identification of *B. bassiana* isolates, Hegedus and Khachatourians (1996) developed a *B. bassiana*-specific PCR-based method. Since many years ago, the ribosomal DNA (rDNA) internal transcribed spacer (ITS) region has been recognised as the key “universal” genetic barcode for fungi. Intragenomic comparisons of high quality genomes of Hypoxylaceae (Xylariales, Ascomycota) revealed a majority of polymorphisms in the ITS regions (Stadler et al., 2020). A recent DNA sequence analysis of the ITS and the elongation factor 1-α (EF1-α) gene of *B. bassiana* has shown that a sampling of globally distributed species complexes combined with morphological and molecular phylogenetic analysis is an efficient strategy for assessing species diversity and the necessary first step in detailing the species evolutionary history and historical ecology (Rehner and Buckley, 2005).

The virulence of *B. bassiana* can be improved by understanding pathogenic pathways. *B. bassiana* infects the host insect by penetrating the cuticle. The insect cuticle, which is made up of a thin outer epicuticle with lipid and proteins and a thick procuticle with chitin and proteins, forms an efficient barrier protecting against invasion by eukaryotic parasites and infection by microorganisms (Moret and Moreau, 2012). By producing proteases first, followed by chitinases, and lipases, *B bassiana* penetrates the insect cuticle through a combination of mechanical pressure from expanding hyphae and enzymatic destruction of the proteins, chitin, and lipids that make up the cuticle (Gillespie et al., 2000). The chitinase gene has been intensively studied for a pest control agent due to its ability to dissolve chitin (Kramer et al., 1993). The entomopathogenic fungus Bbchit1 gene can greatly improve the virulence of transgenic *B. bassiana* against aphids (Fang et al., 2005). *Beauveria* species are utilized as a model system for studying fungal insect pathology and biological insect pest control (Rehner and Buckley, 2005; Imoulan et al., 2016). Spider mites are most common and important polyphagous pests of agricultural ecosystem (Sakunwarin et al., 2003; Pang et al., 2004). Nearly 1,300 species of spider mites are described worldwide, 100 of them are phytophagous and 10 of them are serious agricultural pests. *Tetranychus truncatus* Ehara, a spider mite species, commonly called as a cassava mite, is a significant pest of agricultural crops. It was initially reported in Japan on mulberry (Ehara, 1956). Traditional acaricides are being used to manage the *Tetranychus* group of spider mites, which resulted in the development of resistance to approximately 800 acaricides and 93% of those involve tetranychids (Ullah and Gotoh, 2013). In addition, high fecundity, inbreeding and a very short life cycle result in numerous generations per year, especially in warmer circumstances, which builds the selection pressure for resistance. To solve this issue, numerous studies have demonstrated that *B. bassiana* has insecticidal properties in the form of mycopesticides and commercial endophytic fungi, allowing us to employ it as a natural pesticide alternative (Jaber and Ownley, 2018). Several strains of *B. bassiana* have been developed into a commercial biopesticides of several pests (BotaniGard^®^ES, BotaniGard^®^22WP, Naturalis^®^TNO, and Mycotrol^®^). However, currently there are no registered formulations with CIB&RC (Central Insectiside Board and Registration Committee) against mites in India. Therefore, the present study was undertaken to screen a highly virulent indigenous *B. bassiana* strains against *T. truncatus*.

Commercial bio-pesticides have been recommended in India for the control of a variety of insect pests on different crops, but their use has resulted in lower pesticide efficacy or non-adaptability to Indian agro-climatic conditions. With these considerations, we present a molecular phylogenetic analysis of native *B. bassiana* strains from different regions. We compared and integrated reconstructed phylogenies of three nuclear loci, ITS+ EF1-α + Bbchit1 gene, to study the genetic diversity, population dynamics and co-evolutionary pattern of interaction between *B. bassiana* and its insect host. We also established that *B. bassiana* isolates which may be used to control *T. truncatus* and may be commercialized in future.

## Materials and methods

### Soil and specimen collection and fungus isolation

During 2021, from January to December, fifteen soil samples were collected from seven different locations. Similarly, during 2022, March to December, fifteen fungus-infected insect specimens were collected from five different locations (Table 1). Using the serial dilution approach, *Beauveria* strains were isolated from soil samples. Conidia growing on insect cadavers were transferred onto PDA (Potato Dextrose Agar) plates and grown at 25°C. The isolated fungus colonies were transferred to fresh PDA medium. The purified fungal strains were maintained on PDA slants at 4°C. All *B. bassiana* isolates were deposited at the Indian Type Culture Collection (ITCC) at the Indian Agricultural Research Institute, New Delhi.

**Table 1:** Morphological characters of 30 isolates of *B. bassiana* collected from different locations at 16 DAI on Potato Dextrose Agar (PDA) medium.

Isolate			Phenotypic characters of colony			Spores
Isolates code	Location	Host	Colour	Elevation	Shape	Size (μm)
Bb1	Haryana	Sugarcane borer	white	Flat	Round	2.0 × 1.5
Bb2	Uttarakhand	Paddy stem borer	white	Flat	Round	2.4 × 1.2
Bb3	Karnataka	Brinjal fruit borer	white	Flat	Round	2.0 × 1.8
Bb4	Tamilnadu	Shoot borer	white	Raised	Round	2.0 × 1.5
Bb5	Karnataka	Tomato fruit borer	Orangish white	Flat	Round	1.9 × 1.8
Bb6	Delhi	Tomato fruit borer	white	Raised	Round	1.9 × 1.7
Bb7	Delhi	Cotton bollworm	white	Flat	Round	1.9 × 1.8
Bb8	Tamilnadu	Gram pod borer	white	Flat	Round	1.8 × 1.9
Bb9	Delhi	Chickpea pod borer	white	Flat	Round	1.7 × 1.8
Bb10	Delhi	white grub	white	Raised	oval	2.0 × 1.8
Bb11	Karnataka	White grub	white	Flat	Round	2.1 × 1.9
Bb12	Delhi	Silkworm	white	Flat	Round	1.7 × 1.8
Bb13	Kerala	Mite	white	Raised	oval	2.4 × 1.9
Bb14	Punjab	Soil	white	Raised	Round	1.9 × 1.8
Bb15	Tamilnadu	Diamondback moth	white	Raised	Round	1.8 × 1.8
Bb16	Uttarakhand	Soil	white	Flat	Round	2.1 × 2.0
Bb17	Haryana	Soil	Yellowish white	Flat	oval	2.1 × 1.8
Bb18	Delhi	Soil	Yellowish white	Raised	oval	2.4 × 1.8
Bb19	Delhi	Soil	white	Raised	Round	1.9 × 1.8
Bb20	Delhi	Soil	white	Flat	oval	2.5 × 1.8
Bb21	Delhi	Soil	white	Flat	Round	2.0 × 1.9
Bb22	Karnataka	Soil	white	Flat	Round	1.7 × 1.6
Bb23	Karnataka	Soil	white	Flat	Round	1.8 × 1.9
Bb24	Dehradun	Forest Soil	white	Raised	Round	2.0 × 1.9
Bb25	Delhi	Soil	white	Flat	Round	1.7 × 1.6
Bb26	Tamilnadu	Soil	white	Flat	oval	2.0 × 1.7
Bb27	Karnataka	citrus rust mite	white	Flat	Round	2.0 × 1.8
Bb28	Punjab	Soil	white	Flat	Round	1.9 × 1.8
Bb29	Karnataka	Soil	white	Flat	Round	1.7 × 1.6
Bb30	Karnataka	Soil	white	Raised	Round	2.0 × 1.8

### Morphological observations

PDA slant cultures were transferred to PDA plates and incubated for 14 days at 25°C. Mycelia along with sporulation from the PDA cultures were placed on the clean side in a drop of water and then overlaid with a coverslip for morphological examination. A compound microscope along with camera attachment (Progres capture pro2.7-JENOPTIK) was used to make micro-morphological observations, photomicrographs and measurements. All of these isolates were identified using Humber’s identification key (Humber, 2012).

### Molecular identification

At 25°C, *Beauveria* isolates were grown in 50 ml PD broth. Mycelial biomass was collected on filter paper and rinsed three times in sterile distilled water. Genomic DNA was isolated as described by Rehner and Buckley (2005). The quantity and purity of DNA were measured using a Thermo Scientific NanoDrop Lite Spectrophotometer (Thermo Scientific, Waltham, MA, United States). Similarly, aliquots of PCR products were electrophoresed on a 0.8% agarose gel with ethidium bromide and UV transillumination were used to verify them. Supplementary Table 1 lists the three nuclear gene, i.e., ITS, EF1-α, and Bbchit1primers used to PCR amplify from total DNA of distinct fungal isolates. All PCR reactions were performed in a final volume of 25 μl containing 12.5 μl, 2 × Taq PCR Master Mix (Thermo Scientific), 1 μl of each primer, 1 μl of genomic DNA and 9.5 μl of nuclease-free water. The targeted gene’s PCR conditions were followed exactly as reported (ITS and EF1; Rehner and Buckley, 2005; chitinase; Khemika et al., 2006). The PCR results were run on a 1.8% agarose gel and sequenced using the Sanger technique by outsourcing [Anuvanshiki (OPC) Pvt. Ltd].

### Phylogenetic analysis

The combined 3-gene (ITS+EF1-α + Bbchit1) sequences were used in the phylogenetic analysis of 32 isolates, which contained 30 *B. bassiana* isolates (Bb1 to Bb30), 1 reference strain (*B. bassiana*: KTU-24) and 1 outgroup (*Metarhizium anisopliae*: E6). NCBI nucleotide BLAST analysis was used to compare the retrieved nucleotide sequences with GenBank database. Sequences were constructed, modified, and aligned using BioEdit. BioEdit (version 7.09) and Clustal W were used to perform cluster analyses. In the phylogenetic analysis, ambiguously aligned sites were eliminated, and gaps were treated as missing data. After that, the sequences were artificially aligned and ambiguous areas caused by insertions and deletions (indel) were removed. The gene sequences were concatenated after alignment. MEGA version 10 was used largely for maximum-likelihood (ML) phylogenetic analysis (Swofford, 2002) with the Kimura-two-parameter model, heuristic searches with nearest-neighbor interchange branch switching and 10 random taxon additions were adjusted, and bootstrapping was set to 1,000 replications. TrN + I were the best model test for estimating the data (Posada and Crandall, 1998). The robustness of ML tree topology was confirmed using Minimum Evolution (ME) and Neighbour joining (NJ) with 1,000 bootstrap replications. Tamura-Nei distance was used to implement NJ.

### Bioactivity of *Beauveria bassiana* against *Tetranychus truncatus*

#### *Tetranychus truncatus* culture

Isofemale lines of *T. truncatus* culture was raised on clean mulberry (*Morus alba*) leaves on sterilized foam sheet saturated in water in an insect proof climate control chamber with a temperature of 27 ± 1°C, 12:12 l:D photoperiod and a relative humidity of 65 ± 5%. One to 2 days old females were used for the bioassay.

#### Preparation of conidial suspension of *Beauveria bassiana*

Thirty *B. bassiana* isolates and one commercial formulation (Beveroz: *B. bassiana*, Utkarsh^®^) were grown on PDA medium plates at 25 ± 2°C for 7–10 days until extensive conidiation formed. By flooding the plates with sterile aqueous (0.02%) Tween-80, the conidia were extracted from 2-week-old cultures. To remove any mycelial pieces, the suspension was filtered through sterile muslin fabric (Sasidharan and Varma, 2005). A Neubauer hemocytometer was used to measure conidia concentration under a phase contrast microscope, and the concentration was adjusted to 1 × 10^8^ conidia/mL by diluting with Tween-80 solution. Spreaded the 100 μl of a 1 × 10^8^ conidia/mL suspension onto the surface of PDA Petri plates and assessed conidial viability under compound microscope. The numbers of live and non-viable conidia were counted under a microscope after incubation at 28°C for 24 h. If the germ tube was longer than the diameter of the conidium, it was regarded as germinated (Sevim et al., 2010b).

#### *In vitro* bioassay of *Beauveria bassiana*

By using completely randomized design (CRD) experiment, the pathogenicity of 30 *B. bassiana* isolates from various hosts along with one commercial formulation (Beveroz) was assessed against *T. truncatus* under *in vitro* conditions. Mulberry leaves were cut into 4 cm^2^, both sides of leaf discs were sprayed with 1 ml of 1 × 10^8^ conidia per mL suspensions of 30 collected and one commercial *B. bassiana* isolates using potters tower and allowed to dry in room temperature, after drying leaf discs were placed upside down on half inch damp white foam sheets in a 11 cm disposable Petri dishes. On each plate 30 mites were transferred on to the leaf disc. Each isolate was replicated five times. Control was maintained by spraying water containing 0.02% Tween 80 on both sides of leaf discs. Under stereomicroscope, *even after being touched with a brush or needle, T. truncatus did not move; we can conclude that the mites have dead mites. Like* this, the number of *T. truncatus* mortality data obtained from 31 treatments (30 *B. bassiana* isolates + one commercial formulation- Beveroz treated *T. truncatus*) was recorded under stereomicroscope at 3 DAI, 5 DAI and 7 DAI. Similarly, after 5 DAI and 8 DAI, on the body surface of *T. truncatus* was completely covered by *B. bassiana* mycelium (mycosis); this mycosis data was also recorded under a stereomicroscope.

### Statistical analysis

The *T. truncatus* mortality data obtained from 31 treatments (30 *B. bassiana* isolates + one commercial formulation-Beveroz treated *T. truncatus*) in the study was converted to percentage values. Percentage mortality was corrected by Abbott (1925). The corrected mortality was transformed using the arc-sin transformation, and then examined using one way—analysis of variance (ANOVA). Duncan’s Multiple Comparison Test was used to compare the percentage mortality rates. SPSS 23.0 was used to conduct all statistical analyses (IBM Corp, 2013).

## Results

### Morphological observations

**Figure 1:**
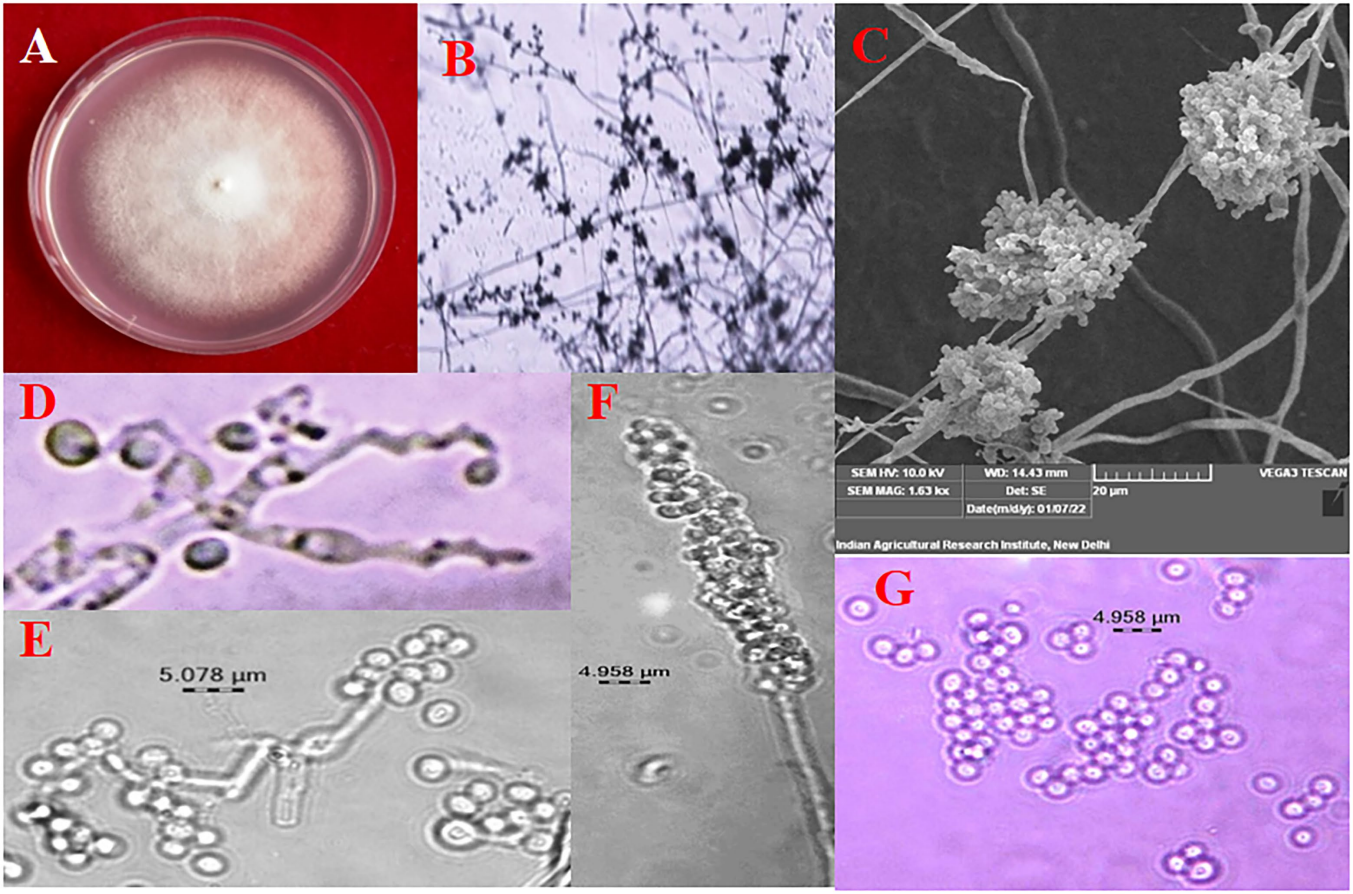
Morphological Characterization of *B. bassiana* **(A)** colony on PDA, **(B)** conidiophore branching under 10X, **(C)** conidial cluster under SEM, **(D)** elongated sympodial rachis, **(E)** and **(F)** conidia production on rachis, **(G)** conidia.

The colony colour, shape, and growth rate of *B. bassiana* isolates were observed macroscopically. Bb5 developed orangish white, Bb17 and Bb18 isolates developed yellowish white colonies, while all other isolates showed white colonies (Supplementary Figures 1A,B). In terms of colony shape, all colonies were circular; no significant among isolates were found. The conidia produced on elongated sympodial rachis of flask shaped phialides were single celled, hyaline and round with a size of 1.7–2.5 × 1.6–1.8 μm (Supplementary Figure 2; Figure 1; Table 1). In terms of mean colony growth rate, the Bb16 isolate outperformed the other 29 isolates (Supplementary Table 2).

### Molecular identification and phylogenetic analysis

**Figure 2:**
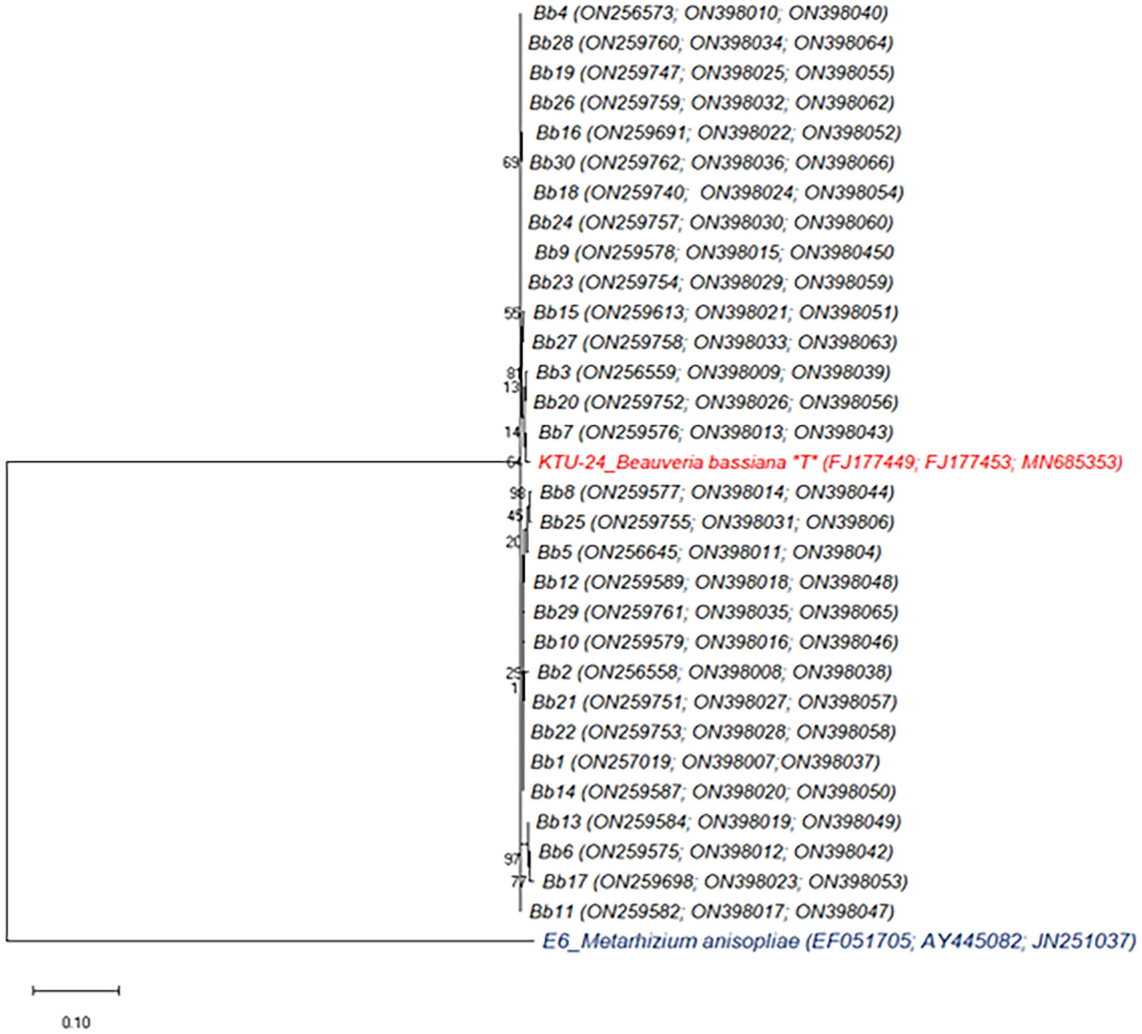
Phylogenetic analysis of *B. bassiana* isolates based on combined partial ITS+TEF + Bbchit1 sequences. The dendrogram was constructed by using Maximum Likelihood method with the kimura 2-parameter model with MEGA 10 version. Isolate representing ex-type material is marked with “T.” Bootstra*p* values shown next to nodes are based on 1,000 replicates. The tree was rooted using isolate *Metarhizium anisopliae* E6 as the outgroup.

Three loci (ITS, EF1-α and Bbchit1) gene amplified products of all the *B. bassiana* isolates were confirmed under gel electrophoresis (Supplementary Figures 3–5). The generated sequences of all the isolates were identified as *B. bassiana* based on NCBI BLAST analysis. All three loci gene sequences of 30 isolates were deposited in Gen Bank and the accession numbers were obtained (Supplementary Table 3). Phylogenetic analyses based on combined partial ITS+ EF1- α + Bbchit1 sequences of *B. bassiana* isolates were performed using the ML (Figure 2), NJ (Supplementary Figure 6) and ME (Supplementary Figure 7) methods of the MEGA version 10 programme and compared with representative sequences from the work of Sevim et al. (2010a). The nucleotides of 2,544, ITS+EF1-α + Bbchit1 gene sites were examined for the 32 samples with base compositions of A = 25%, T = 25%, C = 25%, and G = 25%.

The score of the ML tree was -lnL = 7628.42 log likelihood. ML analytical method executed dissimilar tree topology with NJ and ME (Figure 2). In NJ and ME tree, except six isolates *viz.*, Bb10, Bb11, Bb13, Bb14, Bb19 and Bb20 remaining all other isolates were clustered with *B. bassiana* reference strain (KTU-24) but in case of ML tree, three isolates *viz.*, Bb3, Bb7 and Bb20 clustered very closely with KTU-24 and other showed the higher population diversity. TN93 + G was the best-fit model to explore the dataset (substitution rate matrix R, a = 0.00, b = 0.00, c = 0.07, d = 0.55, e = 4.37). The proportion of invariable sites (I) was 0.00 and gamma distribution shape parameter = 0.22. The pairwise genetic distances among the *B. bassiana* isolates are shown in Supplementary Table 4. The isolates (Bb1 to Bb30) were genetically not closely related (D ranged between 0.003 and 0.036).

### Bioassay of *Beauveria bassiana*

**Figure 3:**
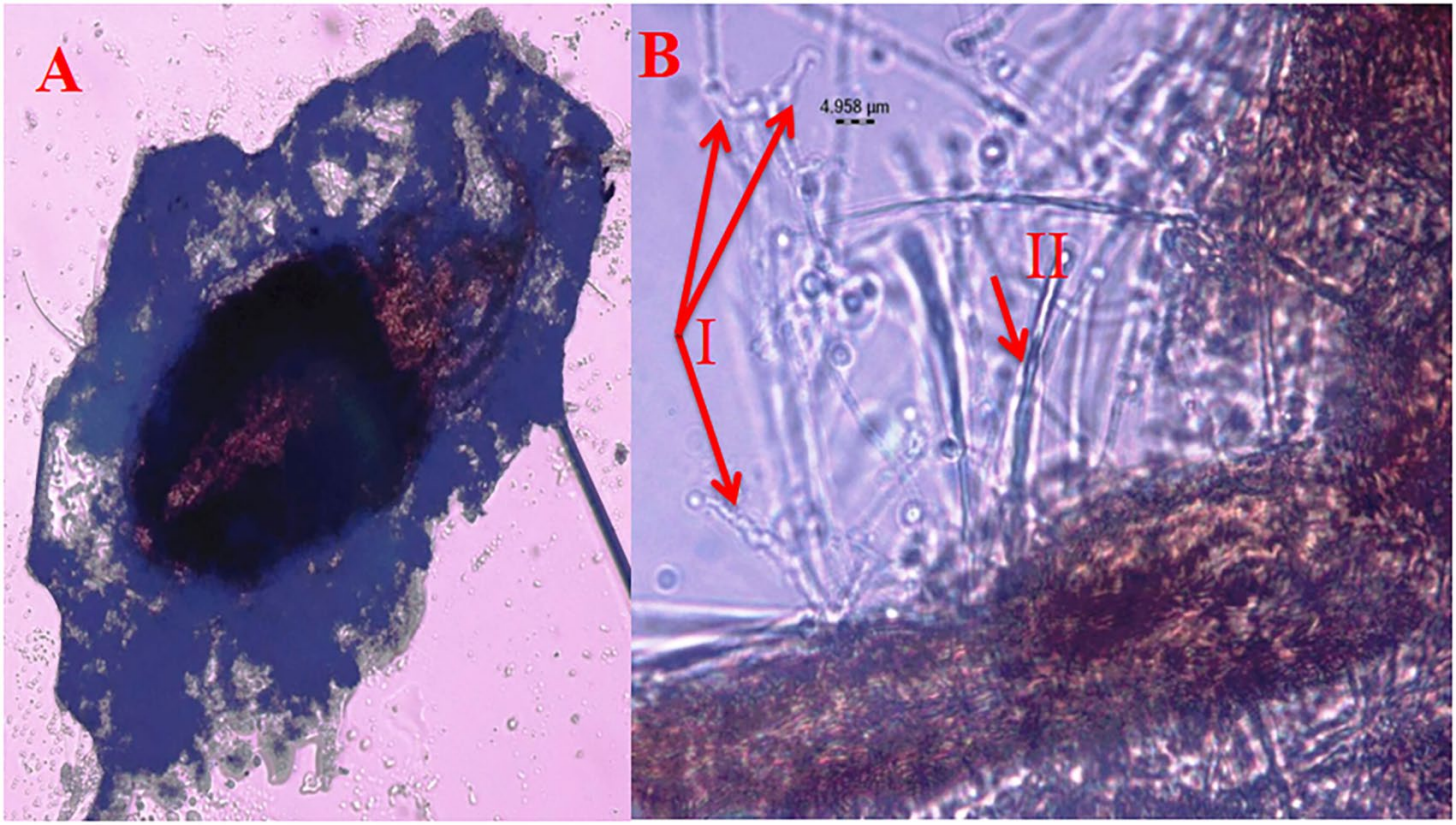
Mortality of *T. truncatus* due to *B. bassiana* **(A)** *T. truncatus* infected with *B. bassiana* under compound microscope, **(B)** details of *B. bassiana* (I.phialide and rachis) and *T. truncatus* (II trichomes).

**Figure 4:**
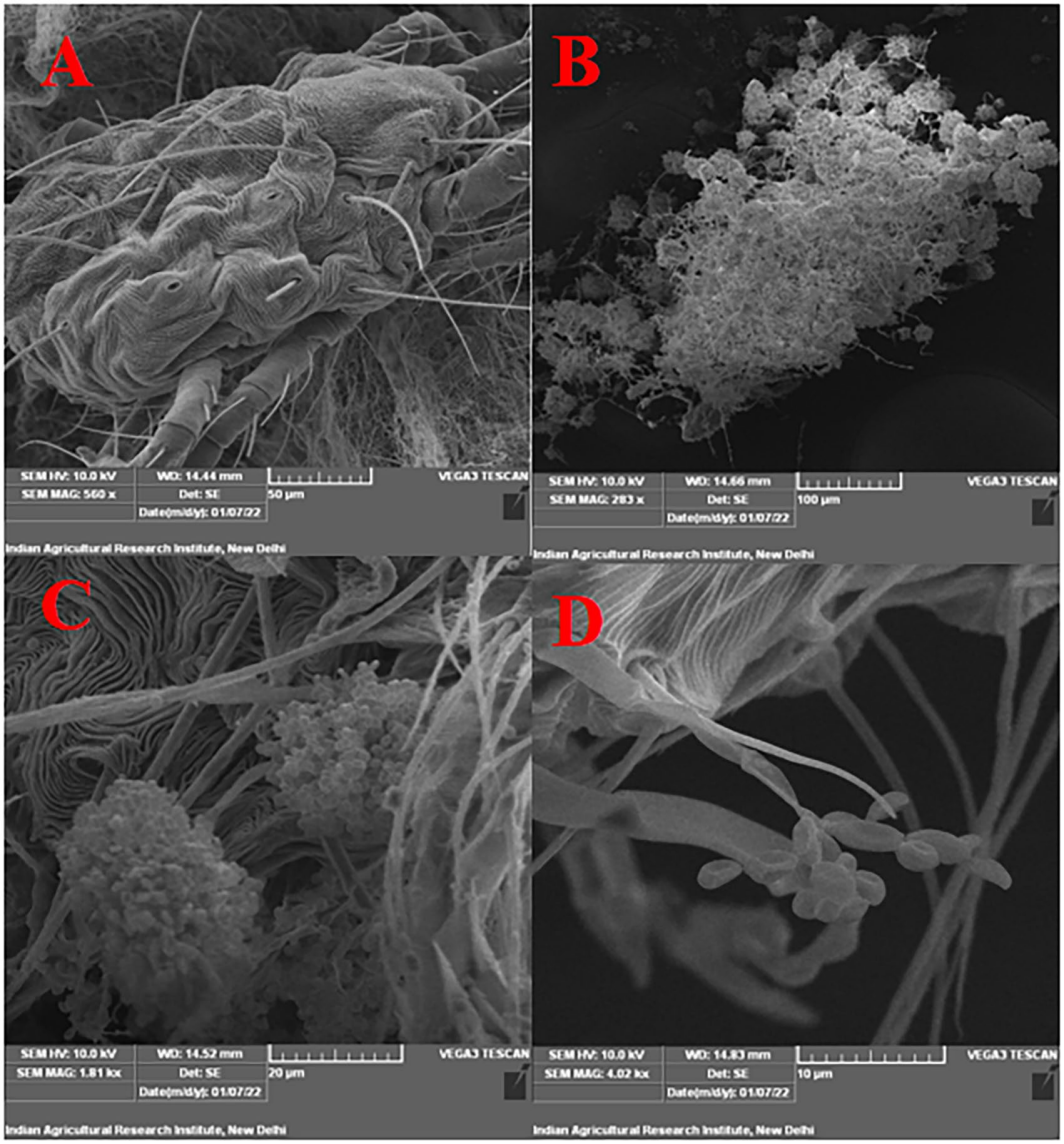
Infection confirmation under SEM **(A)** untreated control (no mortality), **(B)** growth and sporulation of *B. bassiana* on dead *T. truncatus* under 100 μm scale, **(C)** under 20 μm scale, **(D)** under 10 μm scale (phialides and conidia are seen).

**Figure 5:**
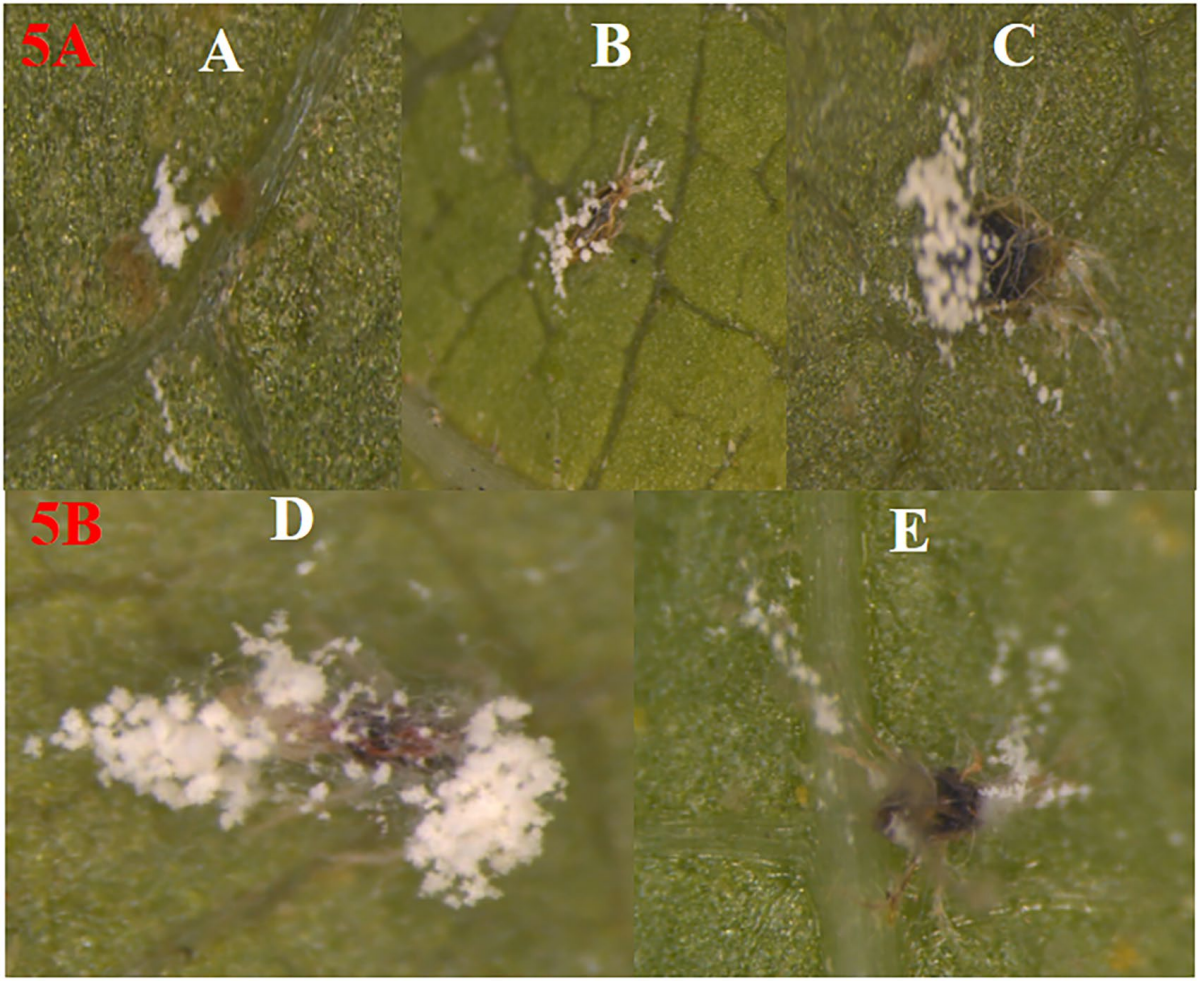
**(5A)** Effect of *B. bassiana* at all growth stages of *T. truncatus.* Mortality of *T. truncatus* at different growth stages due to *B. bassiana* infection **(A)** egg, **(B)** nymph, **(C)** adult. **(5B)** Growth and sporulation differences of **(D)** potential (Bb6) and **(E)** non potential (Bb23) isolates of *B. bassiana*. on *T. truncatus.* Cadaver.

Under laboratory conditions, all 30 *B. bassiana* isolates exhibited different levels of mortality (Table 2) and mycosis (Table 3) of *T. truncatus*. Bb6 isolate collected from tomato fruit borer showed extremely high virulence (97.78%) against *T. truncatus* by killing of mites, followed by Bb15 and Bb12 (96.73 and 94.50%, respectively), which were collected from pupa of diamondback moth and silkworm, respectively, at 7 days after inoculation (DAI) at a concentration of 1 × 10^8^ conidia/mL. After 5–8 DAI, the *T. truncatus* body was covered with mycelium and conidia, which was confirmed by observing under compound microscope (Figure 3) and SEM (Figure 4). The other isolates of *B. bassiana* were significantly less virulent against *T truncatus* including commercial isolate. As a result, Bb6, a native novel isolate of *B. bassiana* was considered as a highly virulent isolate of India against *T truncates*. This isolate was very effective in the mortality of *T. truncatus* even at different stages of growth (Figure 5A). In addition, when compared to other isolates including commercial one, Bb6 and Bb15 isolates produced the greatest mycosis rate of 88% at 8 DAI (highest sporulation), followed by Bb12 (85%; Figure 5B). Mortality rates and mycosis levels in all other isolates ranged from 11 to 89.12% and 0 to 81%, respectively (Supplementary Figures 8, 9). The highly virulent *B. bassiana* isolates grown on mites were re-isolated and maintained for subsequent research work.

**Table 2:** Corrected mortality of *T. truncatus* infected with *B. bassiana* isolates.

**Isolates**	^**^ **% Corrected mortality mean ± SEM**		
	**3 DAI**	**5 DAI**	**7 DAI**
Bb1	*65.09 (52.70) ± 2.34efghi	68.30 (54.60) ± 2.22ef	70.70 (56.53) ± 1.55ef
Bb2	50.88 (45.30) ± 3.74def	65.09 (55.28) ±1.59ef	56.49 (47.78) ± 1.38d
Bb3	64.15 (53.37) ± 2.49fghi	65.09 (53.86) ±1.88ef	65.26 (53.94) ±1.55def
Bb4	18.36 (25.16) ± 1.89b	23.86 (29.15) ±1.41c	28.13 (31.93) ±1.78bc
Bb5	6.49 (13.06) ± 3.51a	11.87 (19.63) ±2.45b	11.93 (19.91) ±1.88a
Bb6	95.67 (80.80) ± 3.92n	96.73 (81.90) ±3.31j	97.78 (84.55) ±3.34i
Bb7	67.37 (55.32) ± 2.36hij	72.87 (58.83) ±2.46 fg	74.85 (60.08) ±2.06 fg
Bb8	5.02 (11.32) ± 3.20a	5.44 (10.41) ±4.37a	6.49 (13.06) ±3.51a
Bb9	60.76 (51.32) ± 2.51efgh	63.98 (53.20) ±1.92ef	67.31 (55.20) ±1.63def
Bb10	30.41 (33.42) ± 1.24bc	33.80 (35.49) ±1.45 cd	34.74 (36.06) ±1.55bc
Bb11	37.02 (37.39) ± 1.97 cd	40.18 (39.26) ±2.14d	41.35 (40.00) ±1.34c
Bb12	90.06 (73.93) ± 4.66mn	93.45 (78.43) ±4.73j	94.50 (79.56) ±4.41i
Bb13	75.09 (60.37) ± 2.60ijkl	76.08 (60.82) ±1.52fgh	77.08 (61.51) ±1.57 fg
Bb14	18.42 (25.31) ± 1.39b	22.81 (28.33) ±2.11c	23.86 (29.11) ±1.77b
Bb15	93.45 (78.67) ± 4.91n	95.56 (80.65) ±3.96j	96.73 (81.90) ±3.31i
Bb16	30.47 (33.40) ± 1.80bc	31.46 (34.02) ±1.89 cd	32.57 (34.75) ±1.40bc
Bb17	66.37 (54.68) ± 2.12ghij	68.54 (55.98) ±1.89ef	70.64 (57.26) ±1.41ef
Bb18	28.19 (31.88) ± 2.27bc	30.47 (33.40) ±1.76 cd	31.40 (33.99) ±1.78bc
Bb19	52.22 (46.31) ± 2.25efg	65.44 (54.17) ±2.79ef	69.59 (56.62) ±1.71ef
Bb20	72.81 (58.61) ± 1.12hik	73.98 (59.45) ±1.71 fg	76.02 (60.80) ±1.60 fg
Bb21	8.71 (15.15) ± 4.16a	11.99 (20.18) ±0.99b	26.37 (29.55) ±8.14b
Bb22	32.46 (34.61) ± 2.15c	33.68 (35.39) ±1.86 cd	35.85 (36.74) ±1.26bc
Bb23	24.04 (29.11) ± 2.52bc	24.97 (29.84) ±1.82c	24.97 (29.84) ±1.82b
Bb24	86.90 (69.26) ± 2.48 m	89.12 (71.59) ±3.10i	89.12 (71.59) ±3.10 h
Bb25	48.65 (44.23) ± 2.90de	57.60 (49.39) ±1.14e	58.71 (50.02) ±0.62de
Bb26	84.85 (67.80) ± 3.18 lm	87.02 (69.31) ±2.34i	88.01 (70.07) ±1.95 h
Bb27	78.30 (62.48) ± 2.18jklm	82.51 (65.82) ±2.83ghi	83.63 (66.62) ±2.66gh
Bb28	80.35 (64.84) ± 2.92klm	82.57 (65.49) ±1.62ghi	83.68 (66.30) ±1.38gh
Bb29	71.64 (57.90) ± 1.49hijk	76.08 (60.75) ±0.88fgh	77.13 (61.46) ±0.84 fg
Bb30	83.63 (66.62) ± 2.66klm	84.68 (67.40) ±2.48hi	85.91(68.27) ±2.03gh
Beveroz	67.30 (55.31) ± 2.36hij	71.80 (58.80) ±2.36 fg	74.75 (60.04) ±2.02 fg
***F ratio	49.01	63.38	54.16

*Figures in parentheses are arcsine transformation values.

** Means followed by the same letter within the same column are not significantly different (*p* < 0.05) by DMRT.

***F and *p* values after square root arcsine transformation.

**Table 3:** Mycosis of *T. truncatus* infected with *B. bassiana* isolates.

Isolates	**% Mycosis mean ± SEM	
	5DAI	8 DAI
Bb1	*43 (40.95) ± 1.48gh	50 (44.99) ± 1.58jk
Bb2	15 (22.37) ±2.47de	17 (24.12) ±1.96 g
Bb3	44 (41.49) ±2.52gh	52 (46.15) ±1.47jk
Bb4	10 (18.20) ±1.57 cd	15 (22.67) ±1.29 fg
Bb5	0 (0.00) ±0.00a	0 (0.00) ±0.00a
BB6	85 (67.33) ±1.29kl	88 (70.06) ±1.95no
Bb7	36 (36.64) ±3.27 fg	53 (46.73) ±2.16jkl
Bb8	1 (2.58) ±2.58a	1 (2.58) ±2.58ab
Bb9	32 (34.33) ±2.08 fg	42 (40.37) ±1.48ij
Bb10	9 (15.39) ±4.25bcd	14 (21.46) ±2.61 fg
Bb11	25 (29.58) ±3.02ef	31 (33.70) ±2.10 hi
Bb12	84 (66.99) ±2.86kl	85 (67.68) ±2.50no
Bb13	63 (52.67) ±2.61ij	69 (56.29) ±2.22 m
Bb14	6 (12.54) ±3.37bc	11(19.07) ±1.82ef
Bb15	88 (71.89) ±4.71 l	88 (71.89) ±4.71o
Bb16	7 (13.41) ±3.83bc	11(17.24) ±4.57def
Bb17	60 (50.82) ±1.86ij	68 (55.74) ±2.56 m
Bb18	4 (7.14) ±4.64ab	6 (9.00) ±5.66bcj
Bb19	41 (39.74) ±2.35gh	51 (45.60) ±2.13 k
Bb20	63 (52.58) ±1.51ij	66 (54.39) ±1.74 l
Bb21	0 (0.00) ±0.00a	5 (9.73) ±4.36bcd
Bb22	10 (17.97) ±2.21 cd	12 (19.83) ±2.30ef
Bb23	7 (11.93) ±4.94bc	7 (11.93) ±4.94cde
Bb24	68 (55.65) ±2.04ij	81 (64.33) ±1.77mno
Bb25	11(18.96) ±2.18 cd	23 (28.58) ±1.33gh
Bb26	73 (59.18) ±3.44jk	78 (62.29) ±2.35 m
Bb27	67 (55.16) ±2.88ij	77 (61.49) ±1.78mn
Bb28	64 (53.16) ±1.12ij	71(57.54) ±1.88 m
Bb29	54 (47.36) ±2.98hi	64 (53.18) ±1.47kl
Bb30	69 (56.26) ±1.80ij	76 (61.08) ±2.92n
Beveroz	35 (35.64) ±3.17 fg	52 (45.73) ±2.15jkl
***F ratio	61.25	66.66

*Figures in parentheses are arcsine transformation values.

** Means followed by the same letter within the same column are not significantly different (*p* < 0.05) by DMRT.

***F and p values after square root arcsine transformation.

## Discussion

The results showed that the agricultural soils and crop pests are important reservoirs of entomopathogenic fungus *B. bassiana*, based on morphological and genetic characterization of this fungus collected from various hosts at different geographical locations. In this regard, it is critical to investigate local conditions in order to identify potential, virulent fungal species. According to Humber’s identification key, all collected 30 isolates were identified as *B. bassiana* based on colony characters and conidial shape and size. All isolates had comparable conidial morphology with hyaline, smooth, and round to oval conidia, which were similar to those previously described (Brady, 1979). Conidia are produced holoblastically from basally inflated conidiogenous cells and grouped amid aerial hyphae as white, spherical clusters. Swollen at the base, the conidiogenous cells stretched into a slender, geniculate or irregularly curved, and denticulate rachis. The isolates morphology matches Glare and Inwood (1998). Phylogenetic analysis of combined three partial sequences of ITS+EF1-α + Bbchit1genes revealed that out of 30 *B. bassiana* isolates, 24 isolates were clustered with *B. bassiana* reference strain (KTU-24) in NJ and ME tree but in case of ML tree, only three isolates *viz.*, Bb3, Bb7 and Bb20 closely clustered with KTU-24. That means ML tree is most appropriate to analyze the population diversity among the *B. bassiana* isolates. The more diverse isolate will give the more chance to prove as potential bio-control agent against insect pests.

The analysis of macroscopic and microscopic features is the basis for traditional morphological identification of fungi (Piontelli, 2015). The most commonly utilized characteristics for determining the species of *Beauveria* are its conidial shape and size (Humber, 2012). Although extremely useful, this identification frequently has limitations in terms of the genetic variability and phenotypic flexibility of the features employed to distinguish species (Paz et al., 2011). Now a days, rDNA genes and ITS allow the investigation of the evolutionary relationships among several groups of entomopathogenic fungi, in addition to morphological identification (Rehner and Buckley, 2005). Therefore, it is crucial to compare the outcomes of morphological characterization and molecular approaches in order to generate more precision in the identification of fungal isolates and study their genetic variability (Hibbett et al., 2011). However, molecular techniques should be utilized to supplement the research to produce a comprehensive, accurate, and appropriately descriptive identification rather than to replace or eliminate the requirement for the usage of classical morphological taxonomy tools (Hyde et al., 2010). Similarly, in the present work in morphological identification, out of 30 *Beauveria* isolates, only three isolates (Bb5, Bb17 and Bb18) had distinct colony shape, color, and shape appearance. But in the case of phylogenetic analysis, out of 30 *B. bassiana* isolates, only three isolates, *viz.*, Bb3, Bb7 and Bb20 clustered closely with KTU-24 (reference strain). So, we can observe that in both cases, not the same three isolates show the same characteristics. Therefore, a technique of morphological and molecular identification for biocontrol agents should be perfect when used as a biological control system against pests (Coates et al., 2002). Pathogenicity related gene identification, in addition to ITS and Ef-1α (housekeeping) genes, aids in determining the virulence nature of pathogenic fungi. Chitin is a key component of inset cuticle, which serves as the first line of defense against pathogens. Over expression of Bbchit1 improved *B. bassiana* ability to digest insect cuticle, resulting in higher insect pathogenicity (Murad et al., 2007). In this investigation, all *B. bassiana* isolates showed amplification at 1047 bp, indicating that all isolates have the chitinase producing gene, which can be correlated with their bio-control ability and expressional analysis. Pallavi (2004) found that entomopathogenic fungal pathogens produced extracellular chitinase against *Helicoverpa armigera*, which supports this finding.

Phylogenetic analysis of combined three partial sequences of ITS+EF1-α + Bbchit1genes revealed that out of 30 *B. bassiana* isolates, 24 isolates were clustered with *B. bassiana* reference strain (KTU-24) in NJ and ME tree but in case of ML tree, only three isolates *viz.*, Bb3, Bb7 and Bb20 closely clustered with KTU-24. That means ML tree is most appropriate to analyze the population diversity among the *B. bassiana* isolates. The more diverse isolate will give the more chance to prove as potential bio-control agent against insect pests. Apart from that, Bb5, Bb17, and Bb18 isolates had distinct colony appearances when compared to the remaining isolates, but.

We also tested all *B. bassiana* isolates for pathogenicity against *T. truncatus*. Most entomopathogenic fungi having acaricidal activities against *T. truncatus* can be attributed to disruption of mite development *via* penetration and subsequent nutrient uptake, according to the earlier studies (Shi and Feng, 2004; Zhang et al., 2014). In the present study, Bb6 isolate seems to have a good potential as a biological control agent against *T. truncatus* as this isolate caused the highest mortality rate and greater mycosis (sporulation) than the other isolates. The findings of this study are similar to Gatarayiha et al. (2012), who were all used entomopathogenic fungi to control mites. The blastospore and aerial conidia efficiency of two *B. bassiana* isolates against *T. urticae* at various stages of development were tested by Al Khoury et al. (2020). 10^9^ blastospores/ml of *B. bassiana* strain exhibited the highest mortality against eggs, motile juveniles, and adults (52, 67.09 and 95.3%, respectively). Similarly, Yun et al. (2017), recorded 77 to 100% mortality rates for *T. urticae* at 7 DAI of aerial conidia, culture filtrate and blastospores of *B. bassiana* 2R-3-3-1 strain against *T. urticae*. Similarly, Yanar et al. (2018), observed mortality rates of *T. urticae* adults from the range of 32.5–72.5% at the end of 72 h incubation period by applying a single dose (5 × 10^6^ conidia ml^−1^) of *B. bassiana* isolates. He also reported mycosis of *T. urticae*, which was ranging between 2.5 and 40.0%. Gatarayiha et al. (2012) reported that mortality caused by 62 *B. bassiana* isolates of *T. urticae* adults ranged between 0.5 and 92.8%; and 23 isolates caused more than 50% mortality. According to De la Rosa et al. (1997), differences in virulence of entomopathogenic fungal isolates are likely due to the presence of enzymes that influence the fungus penetration process. Secondary metabolites, such as toxins like beauvericin found in *B. bassiana*, could also play a role in the observed virulence variation (Roberts and St Leger, 2004). Al Khoury et al. (2019), observed the acaricidal activity of beauvericin against motile stages of *T. urticae* with a concentration of 10, 100 and 1,000 μg/g. In this investigation, Bb6, Bb15, and Bb12 were originally isolated from pest, which caused higher mortality of *T. truncatus* but few isolates isolated from the soil also exhibited more than 50% mortality (Bb17, Bb19, Bb20, Bb24, Bb26, Bb27, Bb28, Bb29 and Bb30 showed 70.64, 69.59, 76.02, 89.12, 88.1, 83.63, 83.68 and 77.13% mortality respectively). The present investigation revealed that there was no correlation exists among mite mortality, geographical location and source of isolation (host). Furthermore, Bb6 isolate caused the highest percentage of mycosis (88%) in mites cadavers. Because sporulation on the host has a key role in fungal spread in the field, isolate Bb6 which showed high percent mortality of *T. truncatus* and also produced high sporulation on dead insect can be used as a microbial pest control agent after field evaluation.

## Conclusion

The local *B. bassiana* isolates were identified using morph-molecular approach and the population diversity was established through phylogenetic analysis of combined ITS+EF1-α + Bbchit1sequences and Bb6 isolate was found as virulent isolate which showed a significant pathogenicity to *T. truncatus*. Further studies are needed to determine the potentiality of Bb6 isolate under field conditions and their molecular bipartite interaction of *B. bassiana* and *T. truncatus*.

## Data availability statement

The original contributions presented in the study are included in the article/Supplementary material; further inquiries can be directed to the corresponding author.

## Author contributions

MC, TP, and DK conceived the review idea and plan. MC did the lab work and statistics and wrote the first draft. MC, TP, DK, LP, SS, and SNB finalize the outline and prepare schematics. LP helped with statistical data analysis and editing of the manuscript. SS and SNB helped in testing the pathogenicity of Beauveria bassiana against *Tetranychus truncatus*. All authors contributed to the article and approved the submitted version.

## Conflict of interest

The authors declare that the research was conducted in the absence of any commercial or financial relationships that could be construed as a potential conflict of interest.

## Publisher’s note

All claims expressed in this article are solely those of the authors and do not necessarily represent those of their affiliated organizations, or those of the publisher, the editors and the reviewers. Any product that may be evaluated in this article, or claim that may be made by its manufacturer, is not guaranteed or endorsed by the publisher.
