# Supplementary material for: “Multilocus sequence analysis for population diversity of indigenous entomopathogenic fungus Beauveria bassiana and its bio-efficacy against the cassava mite, Tetranychus truncatus Ehara (Acari: Tetranychidae)”
Source: Front Microbiol. 2022 Oct 11;13:1007017. doi: 10.3389/fmicb.2022.1007017 (PMC9593087; doi:10.3389/fmicb.2022.1007017)
Supplement: Supplementary file 1 [file Presentation_1.PPTX]

## Slide 1
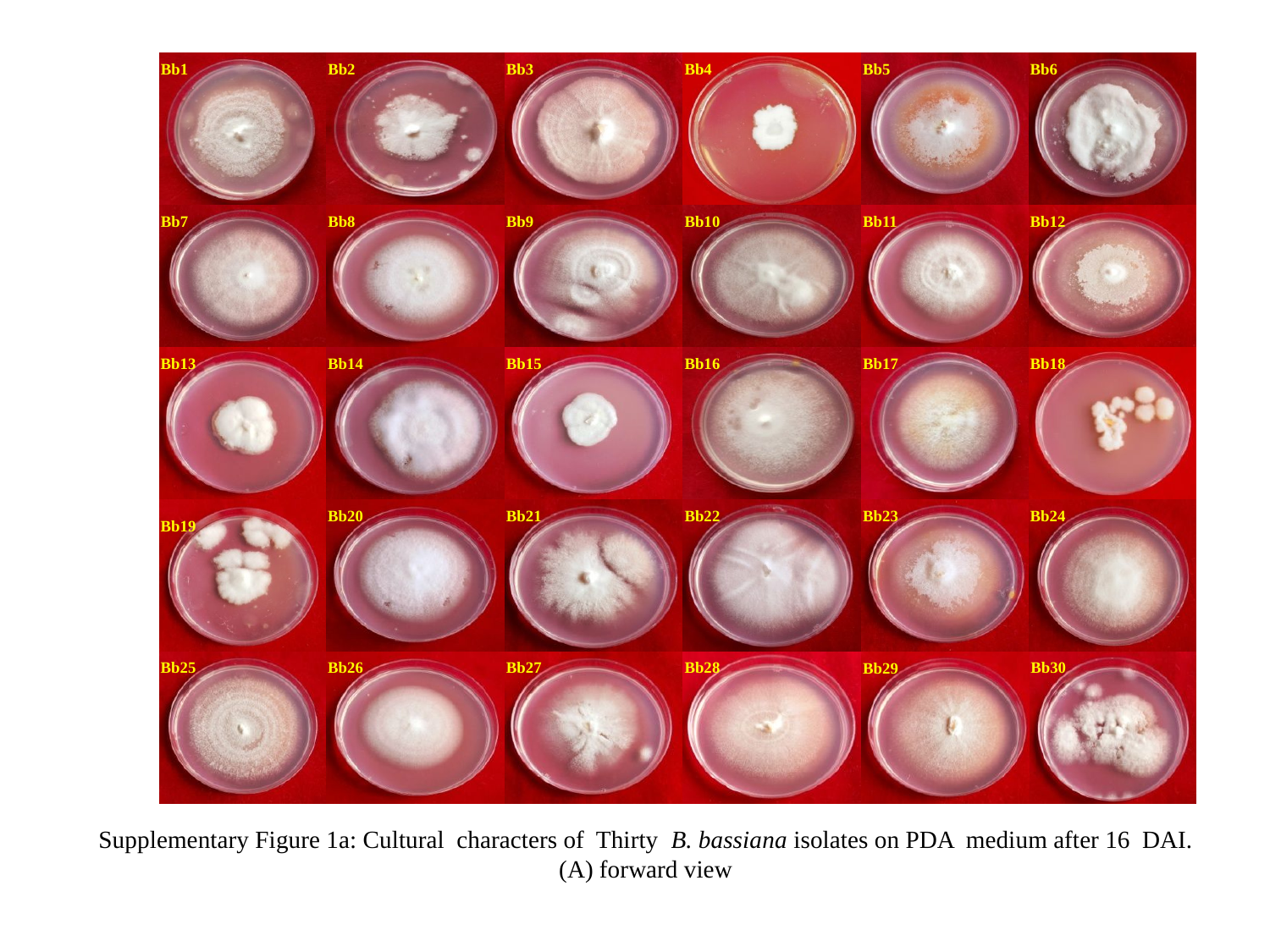

Bb2
Bb3
Bb4
Bb5
Bb6
Bb1
Bb12
Bb7
Bb8
Bb9
Bb10
Bb11
Bb15
Bb18
Bb13
Bb14
Bb16
Bb17
Bb20
Bb21
Bb22
Bb23
Bb24
Bb19
Bb25
Bb26
Bb27
Bb28
Bb30
Bb29
Supplementary Figure 1a: Cultural characters of Thirty B. bassiana isolates on PDA medium after 16 DAI. (A) forward view

## Slide 2
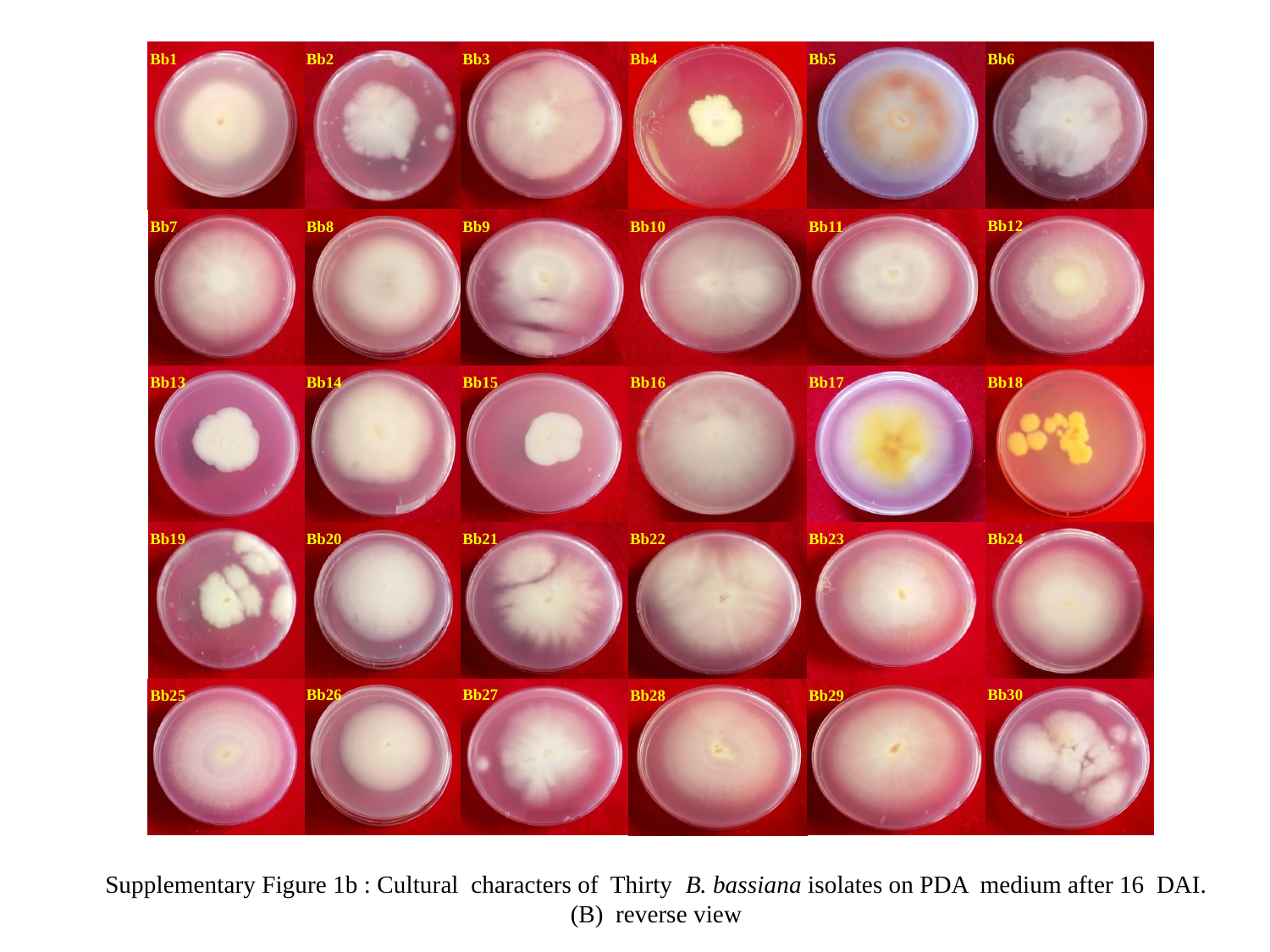

Bb2
Bb3
Bb4
Bb5
Bb6
Bb1
Bb12
Bb7
Bb8
Bb9
Bb10
Bb11
Bb15
Bb18
Bb13
Bb14
Bb16
Bb17
Bb21
Bb19
Bb20
Bb22
Bb23
Bb24
Bb26
Bb27
Bb30
Bb25
Bb28
Bb29
Supplementary Figure 1b : Cultural characters of Thirty B. bassiana isolates on PDA medium after 16 DAI. (B) reverse view

## Slide 3
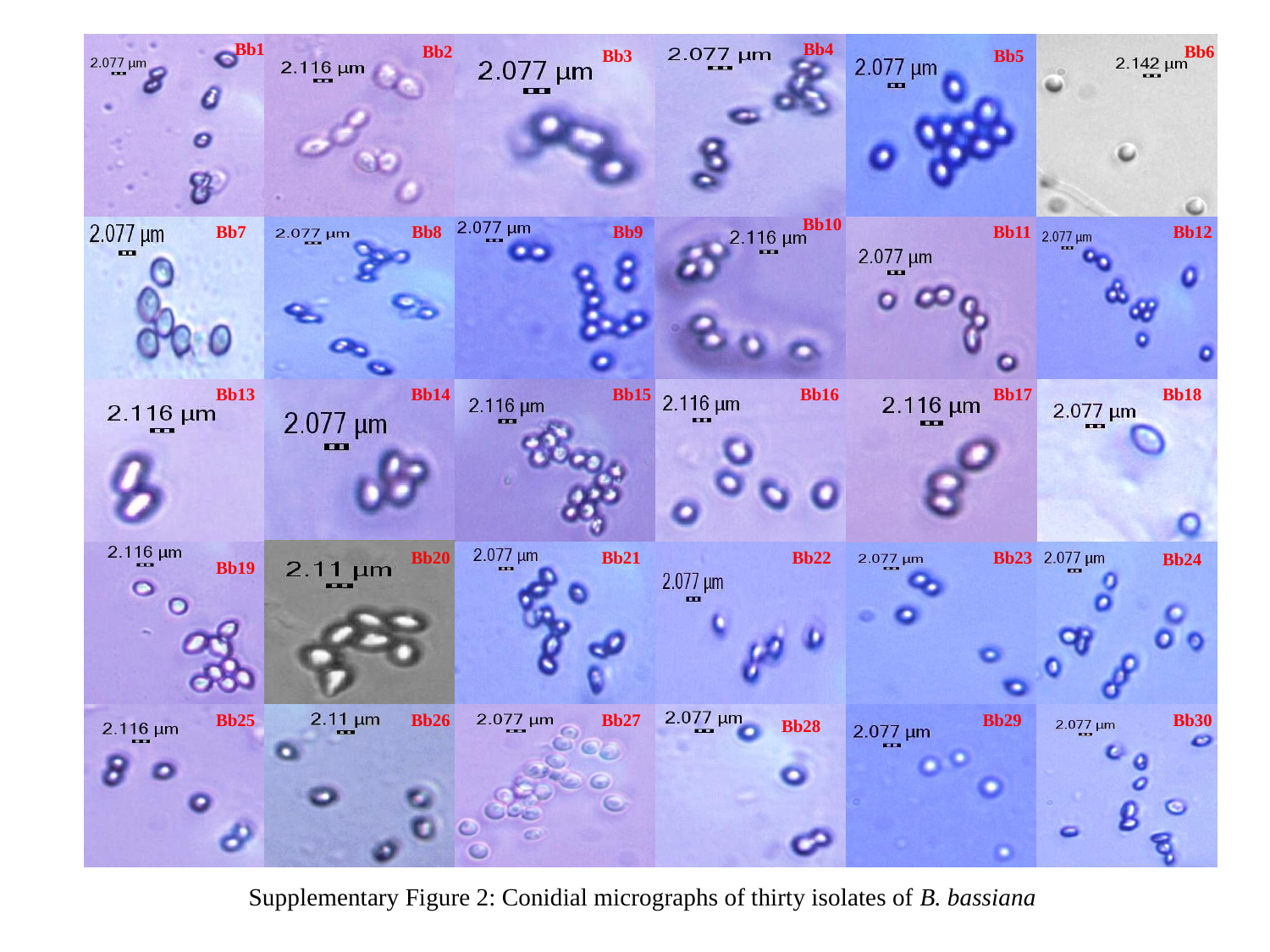

Bb1
Bb4
Bb2
Bb6
Bb3
Bb5
Bb10
Bb7
Bb8
Bb9
Bb11
Bb12
Bb13
Bb14
Bb15
Bb16
Bb17
Bb18
Bb20
Bb21
Bb22
Bb23
Bb24
Bb19
Bb25
Bb26
Bb27
Bb29
Bb30
Bb28
Supplementary Figure 2: Conidial micrographs of thirty isolates of B. bassiana

## Slide 4
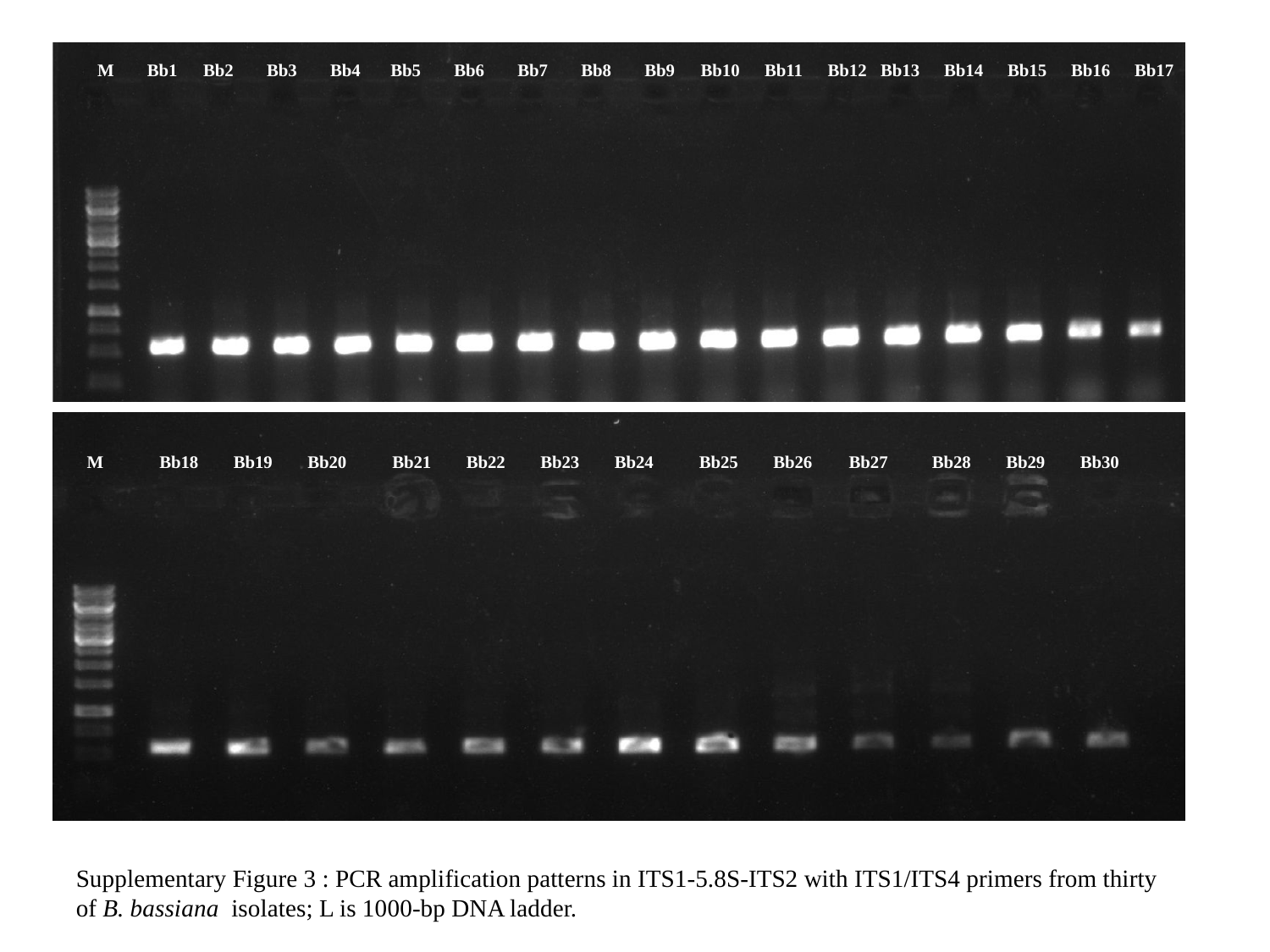

M
Bb1
Bb2
Bb3
Bb4
Bb5
Bb6
Bb7
Bb8
Bb9
Bb10
Bb11
Bb12
Bb13
Bb14
Bb15
Bb16
Bb17
M
Bb18
Bb19
Bb20
Bb21
Bb22
Bb23
Bb24
Bb25
Bb26
Bb27
Bb28
Bb29
Bb30
Supplementary Figure 3 : PCR amplification patterns in ITS1-5.8S-ITS2 with ITS1/ITS4 primers from thirty of B. bassiana isolates; L is 1000-bp DNA ladder.

## Slide 5
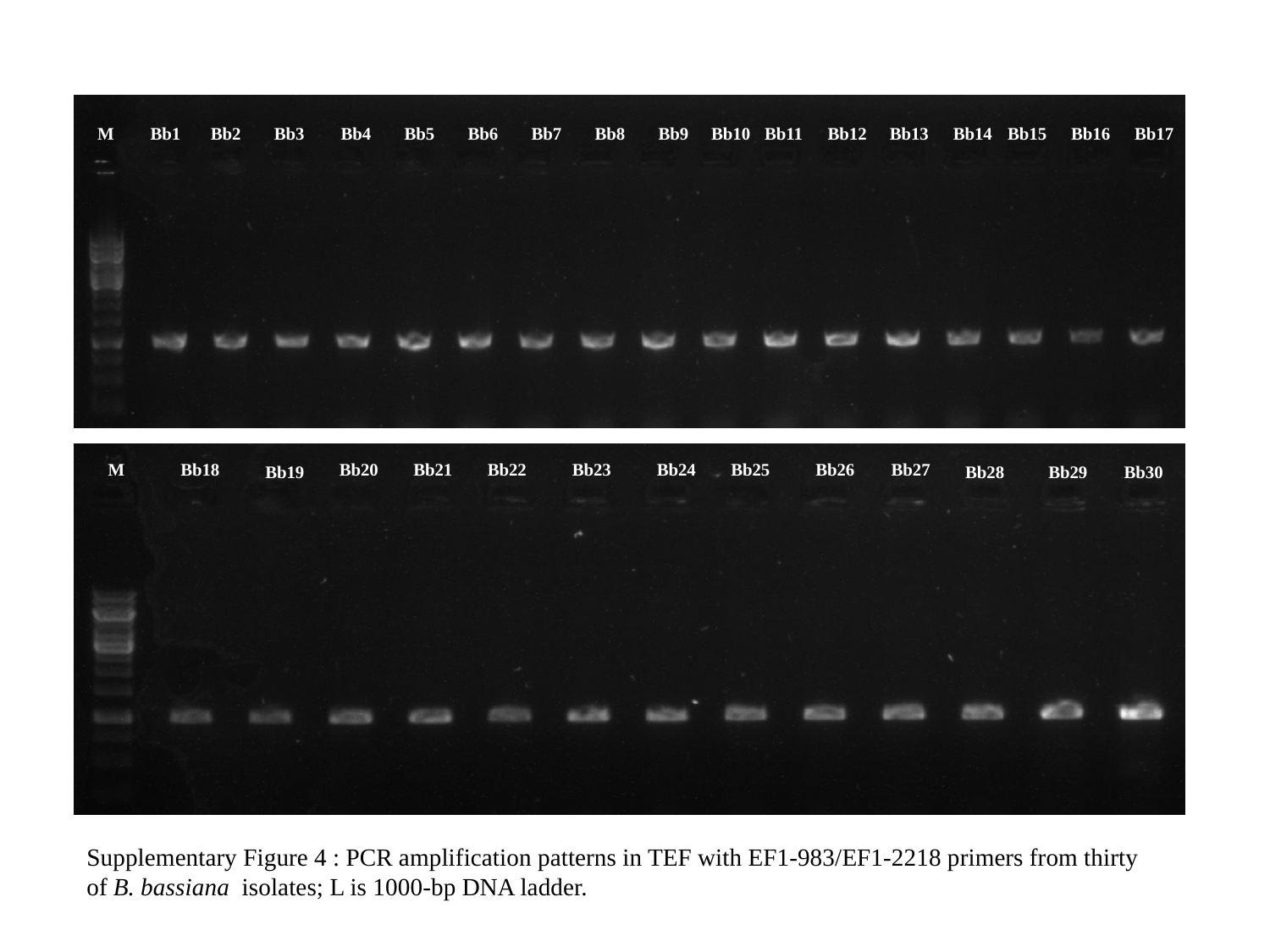

Bb1
Bb2
Bb7
Bb9
Bb11
M
Bb1
Bb2
Bb3
Bb4
Bb5
Bb6
Bb7
Bb8
Bb9
Bb10
Bb11
Bb12
Bb13
Bb14
Bb15
Bb16
Bb17
M
Bb18
Bb20
Bb21
Bb22
Bb23
Bb24
Bb25
Bb26
Bb27
Bb19
Bb28
Bb29
Bb30
Supplementary Figure 4 : PCR amplification patterns in TEF with EF1-983/EF1-2218 primers from thirty of B. bassiana isolates; L is 1000-bp DNA ladder.

## Slide 6
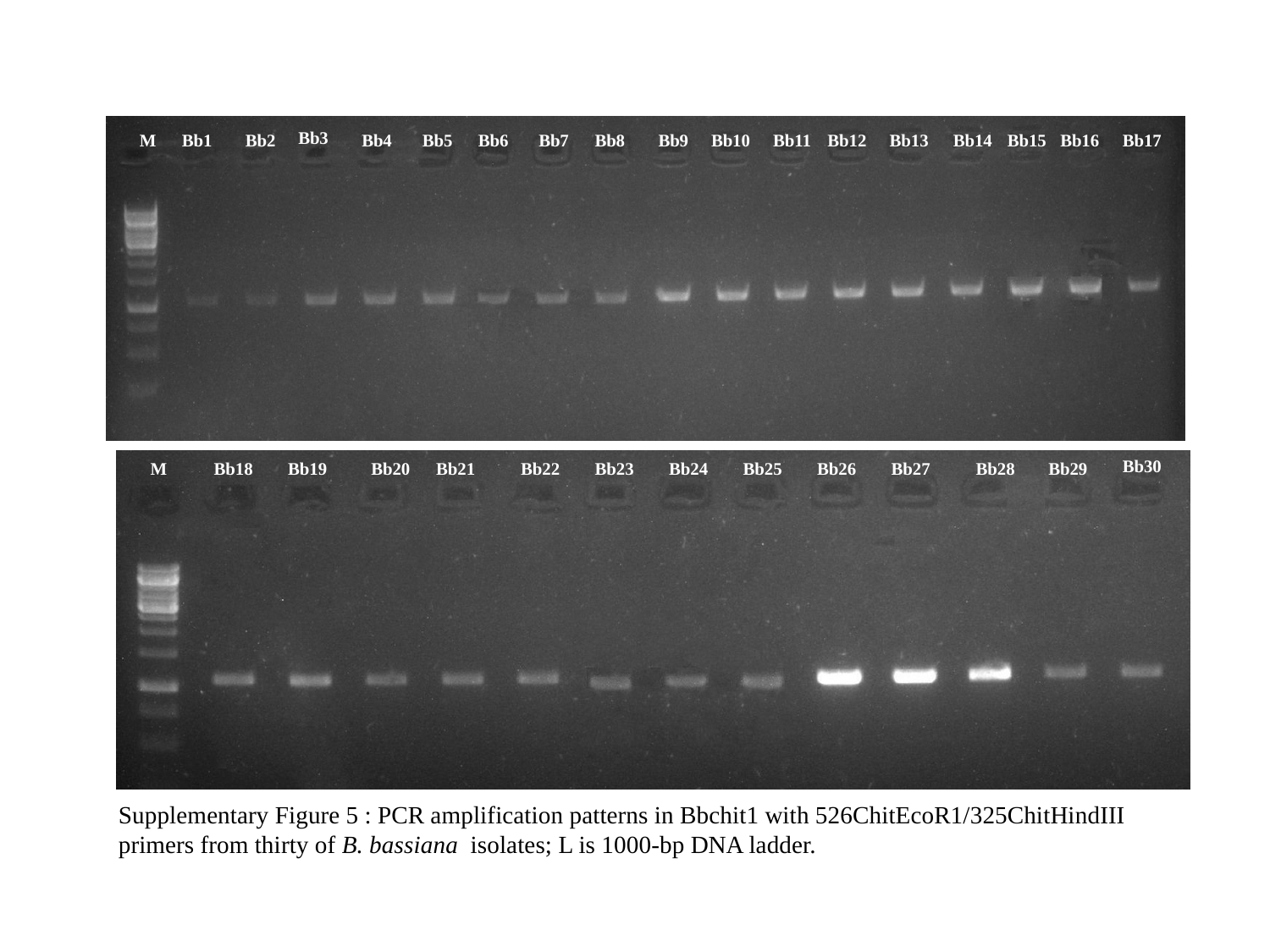

M
Bb4
Bb14
Bb15
Bb3
M
Bb1
Bb2
Bb4
Bb5
Bb6
Bb7
Bb8
Bb9
Bb10
Bb11
Bb12
Bb13
Bb14
Bb15
Bb16
Bb17
Bb30
M
Bb18
Bb19
Bb20
Bb21
Bb22
Bb23
Bb24
Bb25
Bb26
Bb27
Bb28
Bb29
M
Supplementary Figure 5 : PCR amplification patterns in Bbchit1 with 526ChitEcoR1/325ChitHindIII primers from thirty of B. bassiana isolates; L is 1000-bp DNA ladder.

## Slide 7
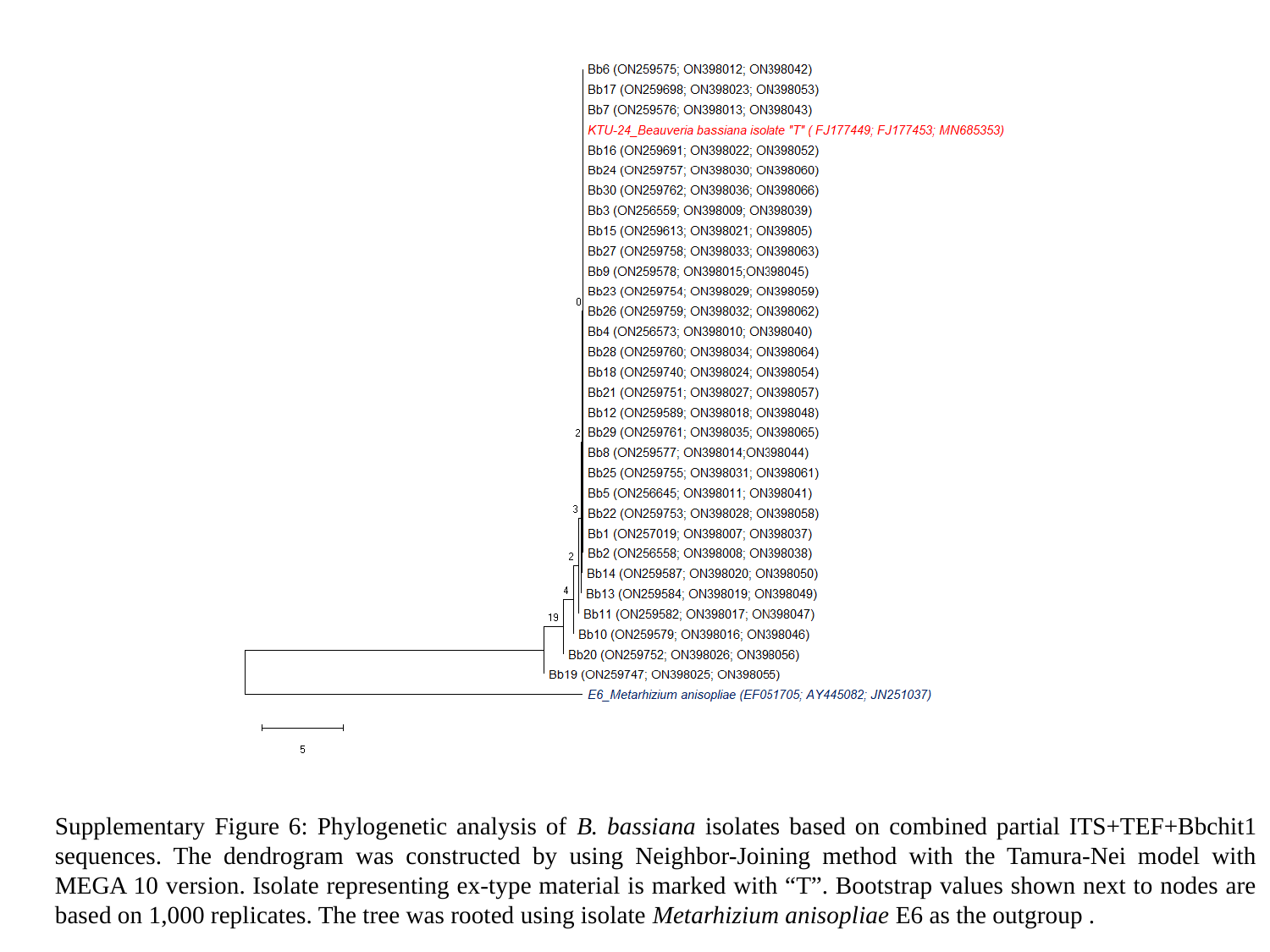

Supplementary Figure 6: Phylogenetic analysis of B. bassiana isolates based on combined partial ITS+TEF+Bbchit1 sequences. The dendrogram was constructed by using Neighbor-Joining method with the Tamura-Nei model with MEGA 10 version. Isolate representing ex-type material is marked with “T”. Bootstrap values shown next to nodes are based on 1,000 replicates. The tree was rooted using isolate Metarhizium anisopliae E6 as the outgroup .

## Slide 8
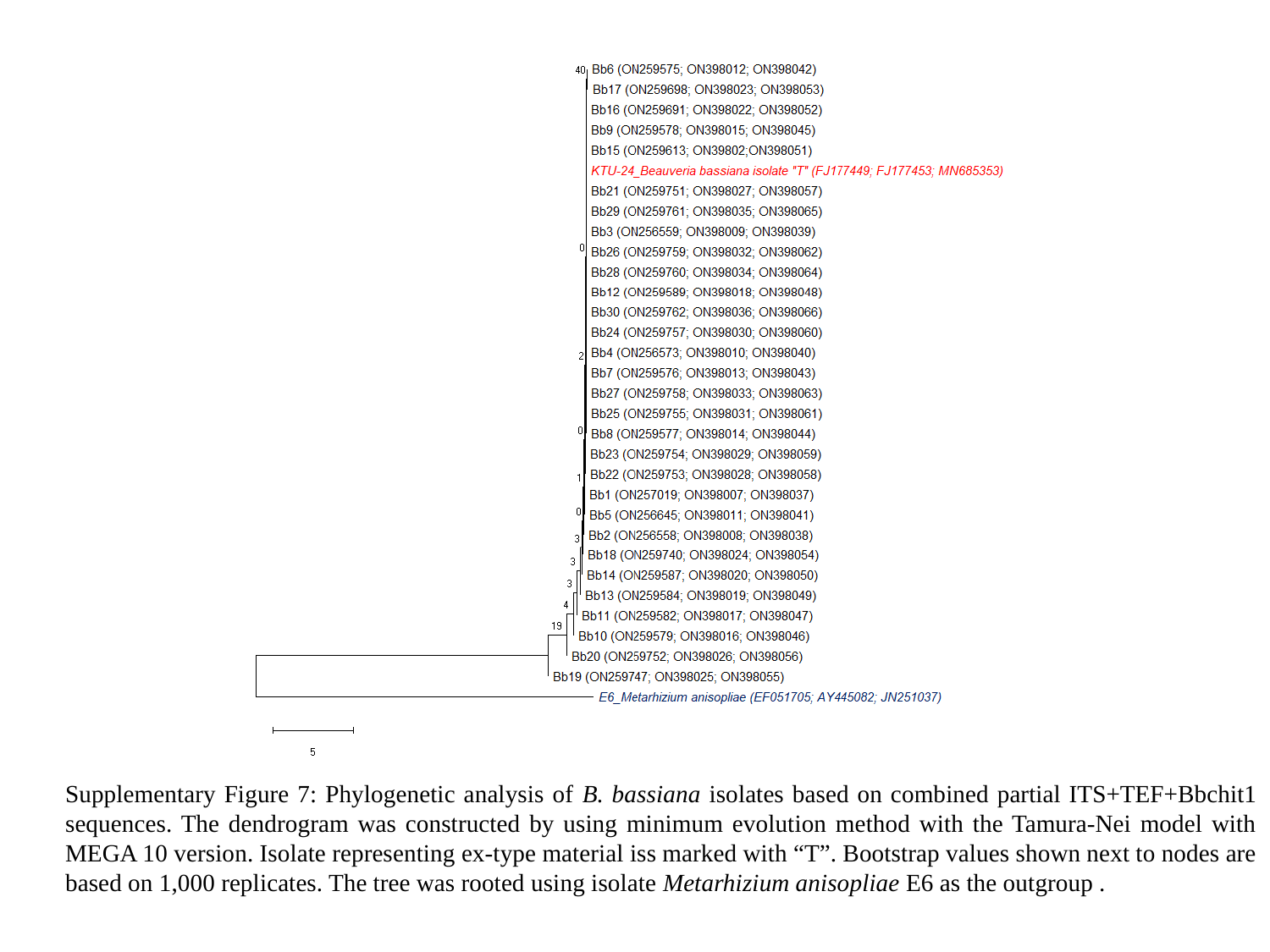

Supplementary Figure 7: Phylogenetic analysis of B. bassiana isolates based on combined partial ITS+TEF+Bbchit1 sequences. The dendrogram was constructed by using minimum evolution method with the Tamura-Nei model with MEGA 10 version. Isolate representing ex-type material iss marked with “T”. Bootstrap values shown next to nodes are based on 1,000 replicates. The tree was rooted using isolate Metarhizium anisopliae E6 as the outgroup .

## Slide 9
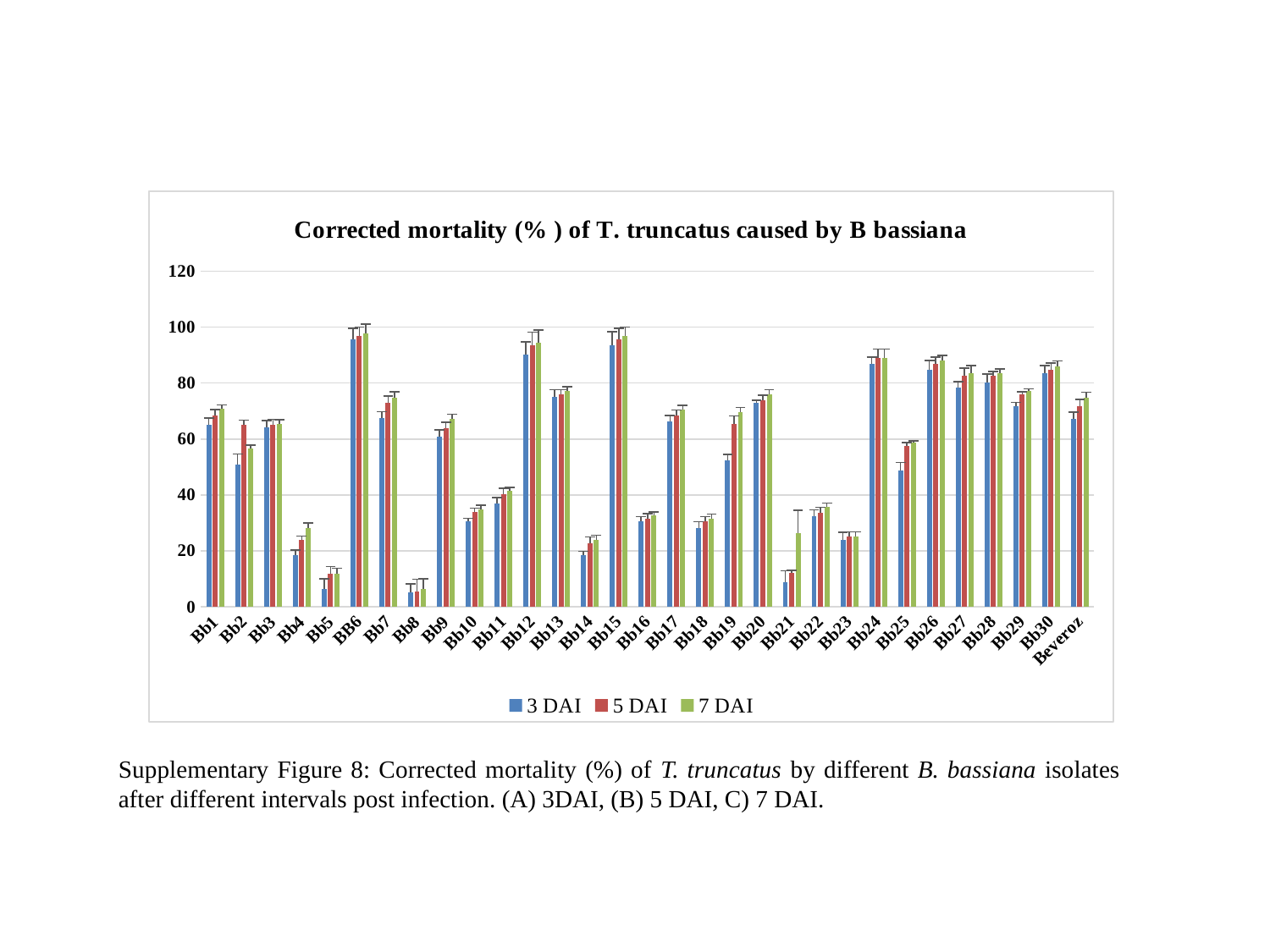

### Chart: Corrected mortality (% ) of T. truncatus caused by B bassiana
| Category | 3 DAI | 5 DAI | 7 DAI |
|---|---|---|---|
| Bb1 | 65.09 | 68.3 | 70.7 |
| Bb2 | 50.88 | 65.09 | 56.49 |
| Bb3 | 64.14999999999999 | 65.09 | 65.26 |
| Bb4 | 18.36 | 23.86 | 28.13000000000003 |
| Bb5 | 6.49 | 11.870000000000006 | 11.93 |
| BB6 | 95.66999999999999 | 96.73 | 97.78 |
| Bb7 | 67.36999999999999 | 72.86999999999999 | 74.85 |
| Bb8 | 5.02 | 5.44 | 6.49 |
| Bb9 | 60.76000000000001 | 63.98 | 67.31 |
| Bb10 | 30.41 | 33.800000000000004 | 34.74 |
| Bb11 | 37.02 | 40.18 | 41.35 |
| Bb12 | 90.06 | 93.45 | 94.5 |
| Bb13 | 75.09 | 76.08 | 77.08 |
| Bb14 | 18.42 | 22.81000000000003 | 23.86 |
| Bb15 | 93.45 | 95.56 | 96.73 |
| Bb16 | 30.47 | 31.459999999999987 | 32.57 |
| Bb17 | 66.36999999999999 | 68.54 | 70.64 |
| Bb18 | 28.19 | 30.47 | 31.4 |
| Bb19 | 52.22000000000001 | 65.44000000000003 | 69.59 |
| Bb20 | 72.81 | 73.98 | 76.02 |
| Bb21 | 8.71 | 11.99 | 26.37 |
| Bb22 | 32.46 | 33.68 | 35.85 |
| Bb23 | 24.04 | 24.97 | 24.97 |
| Bb24 | 86.9 | 89.11999999999999 | 89.11999999999999 |
| Bb25 | 48.65 | 57.6 | 58.71 |
| Bb26 | 84.85 | 87.02 | 88.01 |
| Bb27 | 78.3 | 82.51 | 83.63 |
| Bb28 | 80.35 | 82.57 | 83.67999999999998 |
| Bb29 | 71.64 | 76.08 | 77.13 |
| Bb30 | 83.63 | 84.67999999999998 | 85.91000000000003 |
| Beveroz | 67.3 | 71.8 | 74.75 |Supplementary Figure 8: Corrected mortality (%) of T. truncatus by different B. bassiana isolates after different intervals post infection. (A) 3DAI, (B) 5 DAI, C) 7 DAI.

## Slide 10
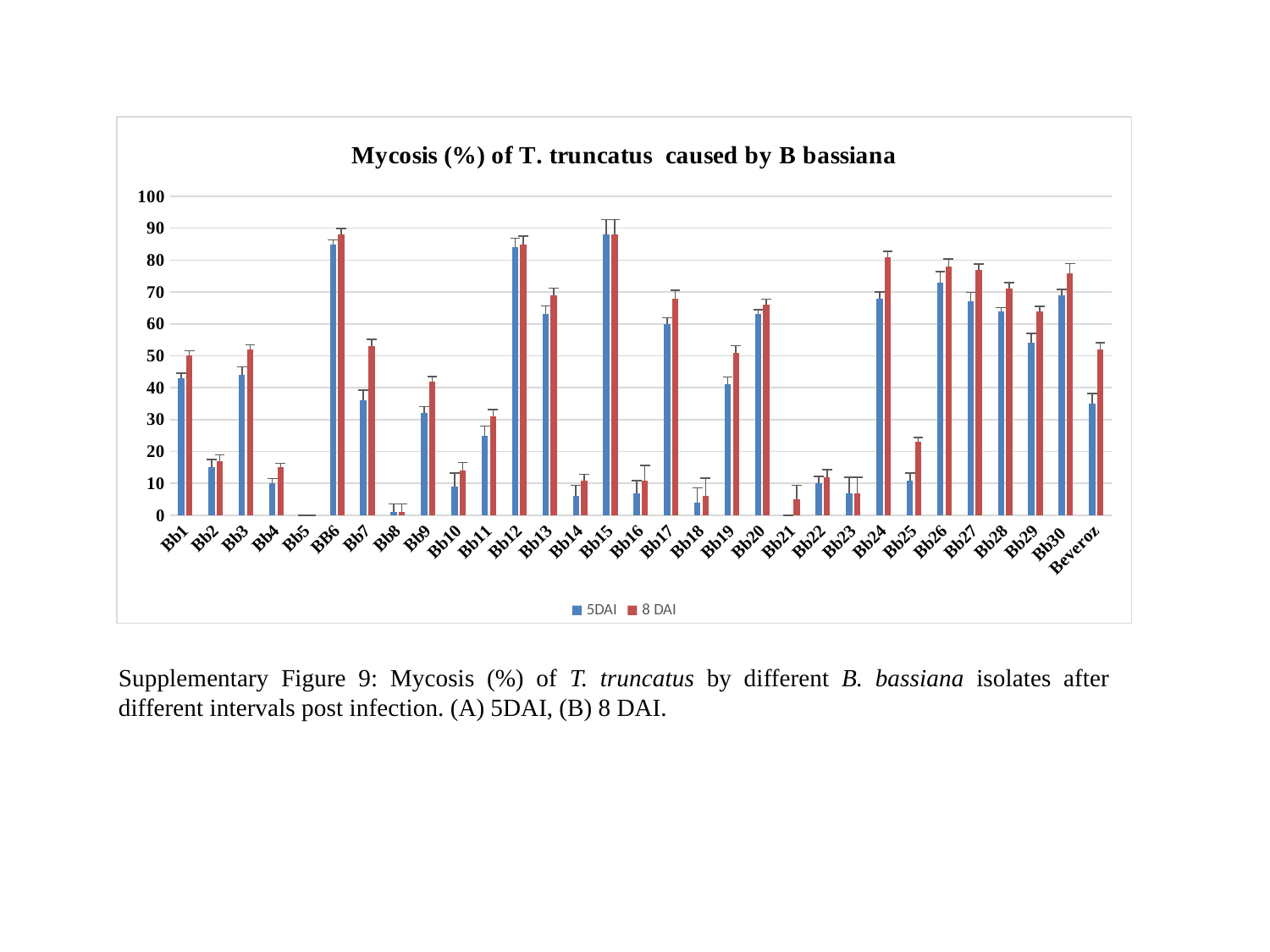

### Chart: Mycosis (%) of T. truncatus caused by B bassiana
| Category | 5DAI | 8 DAI |
|---|---|---|
| Bb1 | 43.0 | 50.0 |
| Bb2 | 15.0 | 17.0 |
| Bb3 | 44.0 | 52.0 |
| Bb4 | 10.0 | 15.0 |
| Bb5 | 0.0 | 0.0 |
| BB6 | 85.0 | 88.0 |
| Bb7 | 36.0 | 53.0 |
| Bb8 | 1.0 | 1.0 |
| Bb9 | 32.0 | 42.0 |
| Bb10 | 9.0 | 14.0 |
| Bb11 | 25.0 | 31.0 |
| Bb12 | 84.0 | 85.0 |
| Bb13 | 63.0 | 69.0 |
| Bb14 | 6.0 | 11.0 |
| Bb15 | 88.0 | 88.0 |
| Bb16 | 7.0 | 11.0 |
| Bb17 | 60.0 | 68.0 |
| Bb18 | 4.0 | 6.0 |
| Bb19 | 41.0 | 51.0 |
| Bb20 | 63.0 | 66.0 |
| Bb21 | 0.0 | 5.0 |
| Bb22 | 10.0 | 12.0 |
| Bb23 | 7.0 | 7.0 |
| Bb24 | 68.0 | 81.0 |
| Bb25 | 11.0 | 23.0 |
| Bb26 | 73.0 | 78.0 |
| Bb27 | 67.0 | 77.0 |
| Bb28 | 64.0 | 71.0 |
| Bb29 | 54.0 | 64.0 |
| Bb30 | 69.0 | 76.0 |
| Beveroz | 35.0 | 52.0 |Supplementary Figure 9: Mycosis (%) of T. truncatus by different B. bassiana isolates after different intervals post infection. (A) 5DAI, (B) 8 DAI.
