# Supplementary material for: “Multilocus sequence analysis for population diversity of indigenous entomopathogenic fungus Beauveria bassiana and its bio-efficacy against the cassava mite, Tetranychus truncatus Ehara (Acari: Tetranychidae)”
Source: Front Microbiol. 2022 Oct 11;13:1007017. doi: 10.3389/fmicb.2022.1007017 (PMC9593087; doi:10.3389/fmicb.2022.1007017)

**Supplementary Table 1:** PCR conditions and primer sequence of ITS+EF-1α+Bbchit1 genes of *B. bassiana*

| **Gene** | **Primers** | **Approximately Product size (bp)** | **Direction** | **Sequence (5’-3’)** | **Annealing (**°C) | **References** |
| --- | --- | --- | --- | --- | --- | --- |
| ITS | ITS 1 | 580 | Sense | TCCGTAGGTGAACCTGCGG | 57 | White et al., 1990 |
|  | ITS 4 |  | Antisense | TCCTCCGCTTA TTGATATGC |  |  |
| TEF | EF1-983F | 1010 | Sense | GCYCCYGGHCAYCGTGAYTTYAT | 55 | Sung et al., 2007 |
|  | EF1-2218R |  | Antisense | ATGACACCRACRGCRACRGTYT |  |  |
| Bbchit1 | 523ChitEcoRI | 1047 | Sense | ACATAGGAATTCATGGCTCCTTTTCTTCAAAC | 56.1 | Khemika et al., 2006 |
|  | 325ChitHindIII |  | Antisense | TACCTAACATGAACATTTAAGCTTTT |  |  |

**Supplementary Table 2:** Mean radial growth rate of *B. bassiana* isolates on PDA medium

| **Isolates** | **Mean growth rate (mm/day) ± SEm** | | | | | |
| --- | --- | --- | --- | --- | --- | --- |
|  | **6DAI** | **8DAI** | **10DAI** | **12DAI** | **14DAI** | **16DAI** |
| Bb1 | *25.33±0.60bcde | 37.83±0.44g | 45.50±0.50a | 59.50±0.29ij | 65.83±0.83j | 65.83±0.83g |
| Bb2 | 24.83±2.74bcde | 31.00±0.58e | 36.00±0.76a | 43.50±0.58c | 45.83±0.44b | 45.83±0.44b |
| Bb3 | 33.67±1.33ghij | 43.33±0.44ij | 53.17±0.60a | 59.83±0.17ij | 62.67±0.33gh | 62.67±0.33f |
| Bb4 | 16.67±0.83a | 23.17±0.93c | 26.33±1.96a | 53.17±1.59g | 53.17±1.59de | 53.17±1.59d |
| Bb5 | 26.50±1.00bcdef | 54.33±0.33jp | 66.67±0.17a | 69.33±0.44nop | 72.50±0.29mno | 72.50±0.29lmn |
| BB6 | 40.83±0.17klmn | 52.53±0.26o | 58.00±0.29a | 69.50±0.29nop | 74.67±0.33p | 74.67±0.33o |
| Bb7 | 28.00±1.61def | 38.00±0.29g | 48.83±0.17a | 58.67±0.44i | 68.33±0.33k | 71.67±0.33jklm |
| Bb8 | 24.00±0.76bcd | 45.83±0.44k | 55.33±0.60a | 61.17±0.60j | 70.50±1.04lm | 74.50±0.29o |
| Bb9 | 39.17±3.00klm | 52.20±0.15cno | 68.17±0.44a | 66.83±0.44lm | 64.83±0.17ij | 71.67±0.33klm |
| Bb10 | 39.00±1.00jklm | 42.50±0.29hi | 51.23±0.43a | 58.67±0.17i | 61.00±0.58fg | 78.50±0.29p |
| Bb11 | 43.50±1.50mno | 51.50±0.50mno | 57.17±0.33a | 65.83±0.44kl | 71.17±0.60lmn | 71.17±0.60jkl |
| Bb12 | 40.83±2.20klmn | 43.17±0.60ij | 52.00±0.29a | 64.83±0.17k | 71.83±0.44lmn | 71.83±0.44klm |
| Bb13 | 22.33±1.09bc | 28.67±0.44d | 60.67±0.79b | 51.50±1.76fg | 51.50±1.76cd | 51.50±1.76cd |
| Bb14 | 30.83±1.42fgh | 37.83±0.17g | 46.83±0.33a | 55.83±0.44h | 69.67±0.17kl | 69.67±0.17ij |
| Bb15 | 30.17±4.34efg | 21.33±0.17b | 36.17±0.73a | 36.50±0.29b | 42.00±0.29a | 42.00±0.29a |
| Bb16 | 36.33±0.88ijk | 50.33±0.33m | 62.50±0.29a | 71.83±0.17q | 78.50±0.29q | 78.83±0.17p |
| Bb17 | 23.00±0.50bcd | 32.83±0.17f | 42.83±0.44a | 51.33±0.73efg | 63.83±0.73hij | 68.50±0.29hi |
| Bb18 | 16.00±1.53a | 20.00±0.58a | 41.83±0.44a | 51.23±0.72ef | 51.23±0.72cd | 51.23±0.72c |
| Bb19 | 22.00±1.53b | 22.67±0.17 | 29.17±0.44a | 31.50±0.76a | 41.83±0.44a | 41.83±0.44a |
| Bb20 | 37.67±1.17jkl | 46.67±1.20k | 55.00±0.58a | 68.83±0.44no | 72.50±0.58mno | 72.50±0.58lmn |
| Bb21 | 31.83±0.44fghi | 32.17±0.33ef | 44.17±0.60a | 47.00±0.29d | 50.50±0.76c | 50.50±0.76c |
| Bb22 | 45.17±1.09no | 48.67±0.33l | 65.70±0.44a | 70.23±0.50opq | 71.83±0.17lmn | 74.83±0.17o |
| Bb23 | 38.50±0.50jklm | 50.17±0.44m | 58.50±0.29a | 61.00±0.58j | 73.33±0.73op | 73.33±0.73mno |
| Bb24 | 26.67±0.88bcdef | 43.00±0.29hi | 47.17±0.60a | 59.33±0.44ij | 63.50±0.76hi | 67.00±0.29gh |
| Bb25 | 35.67±1.01hijk | 44.50±0.29 | 52.07±0.43a | 61.17±0.60j | 70.83±0.60lm | 70.83±0.60jkl |
| Bb26 | 27.67±1.59cdef | 41.67±0.33h | 51.50±0.29a | 59.33±0.44ij | 70.17±0.44kl | 70.17±0.44ijk |
| Bb27 | 24.17±2.49bcd | 31.83±0.17ef | 35.83±0.44a | 49.50±0.29e | 55.00±0.58e | 55.00±0.58e |
| Bb28 | 47.83±1.48o | 50.17±0.44m | 63.67±0.44a | 68.00±0.29mn | 71.00±0.58lm | 71.00±0.58jkl |
| Bb29 | 42.83±2.68lmno | 56.50±0.29q | 64.83±0.17a | 71.00±0.29pq | 74.17±0.44op | 74.17±0.44no |
| Bb30 | 43.17±1.83lmno | 51.00±0.58mn | 56.33±0.17a | 61.00±0.50j | 60.17±0.44f | 62.17±0.60f |
| F ratio | 27.13 | 539.23 | 1.09 | 267.21 | 228.26 | 300.75 |

*Means followed by the same letter within the same column are not significantly different (p<0.05) by DMRT

**Supplementary Table 3:** Gen Bank accession numbers of ITS, EF-1α and Bbchit1 of 30 isolates of *B. bassiana*

| Isolates code | ITS  Gene Bank | EF-1α  Gene Bank | Bbchit1  Gene Bank |
| --- | --- | --- | --- |
| Bb1 | ON257019 | ON398007 | ON398037 |
| Bb2 | ON256558 | ON398008 | ON398038 |
| Bb3 | ON256559 | ON398009 | ON398039 |
| Bb4 | ON256573 | ON398010 | ON398040 |
| Bb5 | ON256645 | ON398011 | ON398041 |
| Bb6 | ON259575 | ON398012 | ON398042 |
| Bb7 | ON259576 | ON398013 | ON398043 |
| Bb8 | ON259577 | ON398014 | ON398044 |
| Bb9 | ON259578 | ON398015 | ON398045 |
| Bb10 | ON259579 | ON398016 | ON398046 |
| Bb11 | ON259582 | ON398017 | ON398047 |
| Bb12 | ON259589 | ON398018 | ON398048 |
| Bb13 | ON259584 | ON398019 | ON398049 |
| Bb14 | ON259587 | ON398020 | ON398050 |
| Bb15 | ON259613 | ON398021 | ON398051 |
| Bb16 | ON259691 | ON398022 | ON398052 |
| Bb17 | ON259698 | ON398023 | ON398053 |
| Bb18 | ON259740 | ON398024 | ON398054 |
| Bb19 | ON259747 | ON398025 | ON398055 |
| Bb20 | ON259752 | ON398026 | ON398056 |
| Bb21 | ON259751 | ON398027 | ON398057 |
| Bb22 | ON259753 | ON398028 | ON398058 |
| Bb23 | ON259754 | ON398029 | ON398059 |
| Bb24 | ON259757 | ON398030 | ON398060 |
| Bb25 | ON259755 | ON398031 | ON398061 |
| Bb26 | ON259759 | ON398032 | ON398062 |
| Bb27 | ON259758 | ON398033 | ON398063 |
| Bb28 | ON259760 | ON398034 | ON398064 |
| Bb29 | ON259761 | ON398035 | ON398065 |
| Bb30 | ON259762 | ON398036 | ON398066 |

**Supplementary Table 4**: Pairwise genetic distances among the thirty isolates of *B. bassiana*


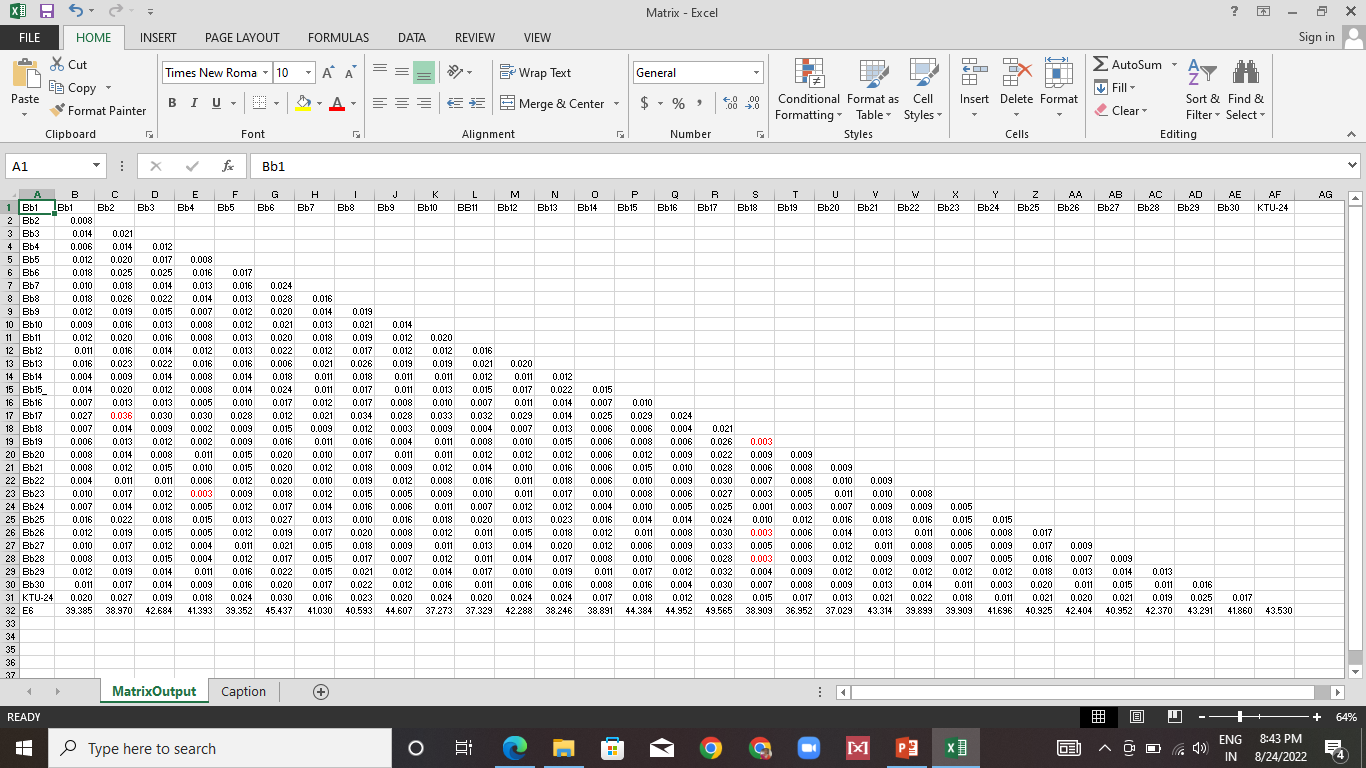

Supplement: Supplementary file 2 [file Data_Sheet_1.docx]
